# SmartBac, a new baculovirus system for large protein complex production

**DOI:** 10.1016/j.yjsbx.2019.100003

**Published:** 2019-02-10

**Authors:** Yujia Zhai, Danyang Zhang, Leiye Yu, Fang Sun, Fei Sun

**Affiliations:** a National Key Laboratory of Biomacromolecules, CAS Center for Excellence in Biomacromolecules, Institute of Biophysics, Chinese Academy of Sciences, Beijing 100101, China; b School of Life Sciences, University of Chinese Academy of Sciences, Beijing, China; c Center for Biological Imaging, Core Facilities for Protein Science, Institute of Biophysics, CAS, Beijing, China

**Keywords:** Baculovirus system, Cre recombination, Gibson assembly, Polyprotein strategy, Protein complex production

## Abstract

Recent revolution of cryo-electron microscopy has opened a new door to solve high-resolution structures of macromolecule complexes without crystallization while how to efficiently obtain homogenous macromolecule complex sample is therefore becoming a bottleneck. Here we report SmartBac, an easy and versatile system for constructing large-sized transfer plasmids used to generate recombinant baculoviruses that express large multiprotein complexes in insect cells. The SmartBac system integrates the univector plasmid-fusion system, Gibson assembly method and polyprotein strategy to construct the final transfer plasmid. The fluorescent proteins are designed co-expressed with the target to monitor transfection and expression efficiencies. A scheme of screening an optimal tagged subunit for efficient purification is provided. Six large multiprotein complexes including the human exocyst complex and dynactin complex were successfully expressed and purified, suggesting a great potential of SmartBac system for its wide application in the future.

## Graphical abstract

**Figure:**
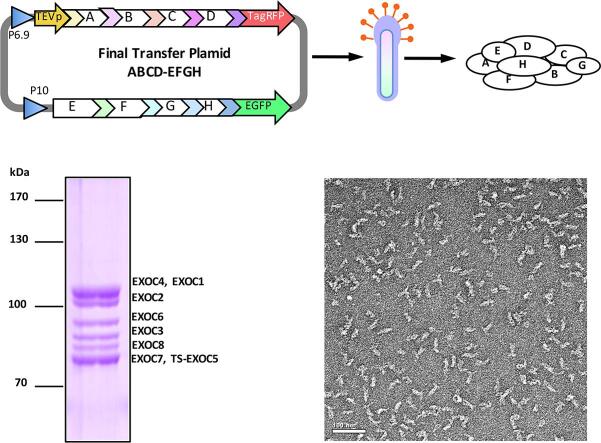

## 1 Introduction

With the rapid development of single-particle cryo-electron microscopy (cryo-EM), more and more macromolecular machineries structures, e.g. spliceosome (Nguyen et al., 2016, Yan et al., 2015a), ryanodine receptor (des Georges et al., 2016, Wei et al., 2016a, Yan et al., 2015b), anaphase promoting complex (Chang et al., 2015), light harvest complex (Wei et al., 2016b) and mitochondrial respirasome (Gu et al., 2016, Letts et al., 2016), have been solved to near-atomic resolution, which have been waiting for many years. Since cryo-EM does not need crystals, many macromolecular complexes that are difficultly crystallized are now ready for structural studies; thus, more and more structural biology laboratories are becoming interested in studying the structures of large macromolecular complexes. However, how to obtain enough highly purified specimen suitable for cryo-EM is therefore becoming a new bottleneck, which has restricted the wide application of cryo-EM technology.

One method for obtaining large protein complexes is to extract them from biological tissues. However, this method is not very suitable for low-abundance samples. In addition to consuming large amounts of reagents, protein extraction usually produces samples with low yield and purity. And even for high-abundance samples, it is still difficult to prepare and purify mutant proteins for functional studies. Thus, recombinant expression of protein complexes is more commonly preferred.

The baculovirus expression system (BVES) is a powerful tool for recombinant protein production (Jarvis, 2009) because it is safe, high expression levels can be achieved, and post-translational modifications can be incorporated. The most common baculovirus used for gene expression is AcMNPV (*Autographa californica* multiple nuclear polyhedrosis virus), which has a large, circular double-stranded DNA genome (about 130 kb) that can accommodate very large exogenous DNA fragments (Jarvis, 2009). However, it is not easy to introduce foreign genes by conventional molecular cloning methods due to the large size of AcMNPV genome. Therefore, researchers have modified the AcMNPV genome to allow effective foreign gene insertion by site–specific transposition or homogenous recombination (Hitchman et al., 2012, Luckow et al., 1993). The widely-used Bac to Bac baculovirus expression system is one successful example (Jarvis, 2009).

Three strategies are commonly used to overexpress multiprotein complexes by baculovirus expression system in insect cells. In the first strategy insect cells are infected with multiple types of baculoviruses, each of which carries one or two gene expression cassettes (GECs). This strategy, which involves molecular cloning, is relatively simple and has been successfully applied by many research groups using the pFastBac series vectors (Birnbaum et al., 2014, Chung et al., 2006, Hu et al., 2003, Kee et al., 2010, Wan et al., 2017, Yoo et al., 2009). However, when multiple types of baculoviruses are used, the total number of infectious viruses added to the expression culture should be comparable to the upper limited number of the single baculovirus infection. This will inevitably lead to a lower protein expression level. In addition, the expression levels of the individual subunits are often imbalanced, which would result improper complex assembly.

The second strategy used to express multiprotein complexes is to construct a transfer plasmid carrying multiple GECs. The commercial pFastbac-Dual vector features two promoters for expression of two proteins simultaneously. Similar triple or quadruple expression vectors have also been built using traditional molecular cloning methods (Belyaev and Roy, 1993). The MultiBac system generates multi-GEC donor and acceptor vectors from junior plasmids carrying individual GECs by homing endonuclease-based multiplication module (Berger et al., 2004). Then the final transfer plasmid is produced by Cre-mediated recombination between the donor and acceptor (Berger et al., 2004). Recently, the biGBac method enabled rapid multiple GECs assembly for large multiprotein complexes expression by a modular “Mix and Match” approach (Weissmann and Peters, 2018, Weissmann et al., 2016). Yet the problem of unequal subunit stoichiometry still exists.

The third strategy is the polyprotein strategy that has been used by coronaviruses to produce multiple functional nonstructural proteins (nsps), which are involved in the assembly of the replication-transcription complex (RTC) that catalyzes viral replication and transcription (Bartlam et al., 2005). The nsps are encoded in open-reading frame 1a (ORF1a) and ORF1b and synthesized initially as two large polyproteins, pp1a and pp1ab. During or after synthesis, these polyproteins are cleaved by virus-encoded proteases into 16 nsps (Ziebuhr et al., 2000). These nsps, together with other viral proteins and possible cellular proteins, assemble into a membrane-bound RTC (Sawicki et al., 2007). This strategy has been recently exploited to express protein complexes using baculovirus system (Nie et al., 2014, Vijayachandran et al., 2011). By this strategy, individual subunits are separated by protease cleavage sites and expressed as a long polyprotein. In vivo processing of the polyprotein allows the proper assembly of the multiprotein complex. This method is very good for balancing expression levels and achieving the correct subunit stoichiometry (Vijayachandran et al., 2011). However, when expressing a very large multiprotein complex, the DNA fragment encoding the polyprotein is very long. It is usually not easy to build such a large transfer plasmid in the average laboratory. In addition, with increasing gene length, gene synthesis becomes more expensive and time-consuming. Other potential problems include inefficient virus amplification and instability of the recombinant baculovirus that is due to the insertion of a large foreign gene.

There are several other considerations that need to be taken into account when using BVES to express large multiprotein complexes. First, the transfer vectors carrying genes of multiple subunits need to be designed for easier molecular cloning. Considering the increased difficulty of molecular cloning with larger constructs (Carter and Shieh, 2010), vectors that allow direct selection for positive transformants are welcome. Second, one tagged subunit is often used to purify entire multiprotein complexes. However, due to the lack of prior knowledge, we usually have to screen the optimal tagged subunit that can yield the most efficient purification of the complex. An efficient experimental scheme is needed to decrease the labor of building these screening vectors. Third, it is also important to quickly determine whether the virus amplification is successful and the expression level is sufficient because insect cell expression systems are more time-consuming compared to *Escherichia coli* (*E. coli*) expression systems. The sooner the problems are identified, the faster they can be solved.

To overcome the above problems in expressing recombinant multiprotein complexes, here we developed SmartBac, a simple and versatile vector system, which combines the advantages of the three strategies described above. To simplify vector construction and obtain more homogenous samples, the polyprotein strategy and Gibson assembly were used to construct the transfer plasmid. The design of a LacZ-alpha cassette allows easier selection of positive recombinants with large DNA inserts by blue-white screening. A univector plasmid-fusion system (UPS) strategy was designed and incorporated into our vector system (Liu et al., 1998). Thus the final construction of large transfer plasmids can be achieved by Cre-loxP site-specific recombination between donor and acceptor. The co-expression of EGFP and tagRFP in the SmartBac system provides real-time visualization of transfection and expression. In addition, the SmartBac system provides a convenient workflow to screen the best tagged subunit for the subsequent optimal purification. Using SmartBac system, we have expressed various large multiprotein complexes, including the human exocyst complex and dynactin complex. We expect this system will aid structural and functional studies of large multiprotein complexes in the future.

## 2 Results

### 2.1 SmartBac vectors

In order to overcome the difficulties in building large plasmids with conventional cloning methods, we incorporated the broadly applicable UPS strategy (Liu et al., 1998) into our SmartBac vector system. Briefly, this strategy uses Cre–loxP site-specific recombination to catalyze fusion between the univector (donor) and host vector (acceptor). The kanamycin-resistant donor has a conditional R6Kγ origin of replication that allows its propagation only in bacterial hosts expressing the pir gene, which encodes the essential replication protein π (Filutowicz et al., 1985, Metcalf et al., 1994). Selection for the UPS recombination products is achieved by selecting for kanamycin resistance (Kan^R^) after transformation into a pir- strain; the Kan^R^ gene in the donor vector can be expressed in a pir-background only when covalently linked to an acceptor that has a functional origin of replication (oriColE1) (Liu et al., 1998). This strategy has been successfully used in MultiBac system (Berger et al., 2004) and is advantageous for the preparation of large plasmids and their mutants due to the relatively small sizes of the donor and acceptor vectors.

**Fig. 1:**
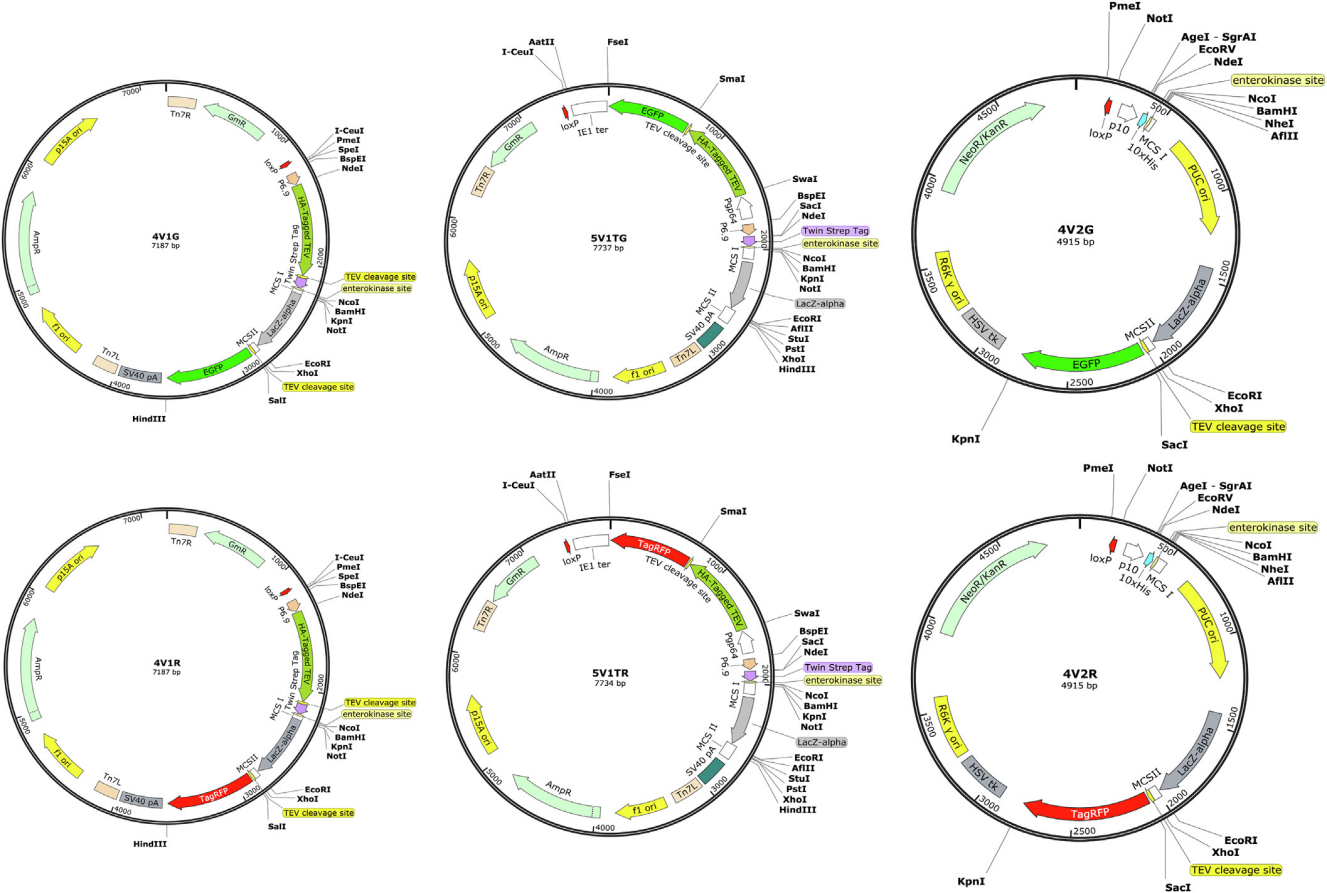
SmartBac vector maps. The SmartBac system includes four acceptor plasmids (4V1G, 4V1R, 5V1TG and 5V1TR) and two donor plasmids (4V2G and 4V2R). Vector maps were produced by SnapGene Software (http://www.snapgene.com/).

We designed four acceptor plasmids (4V1G, 4V1R, 5V1TG and 5V1TR) and two donor plasmids (4V2G and 4V2R) in SmartBac system (Fig. 1, see also Supplementary Materials and Methods S1). The acceptors can recombine with the donors via Cre-LoxP site-specific recombination. The acceptors harbor a p15A origin of replication that allows propagation in common cloning strains of *E. coli* at a low copy number, which is better for the stability of large plasmids. The acceptors also contain resistance markers for ampicillin and gentamycin and flanking mini-Tn7 elements for the generation of recombinant baculoviruses.

Transgene expression in infected insect cells is driven by the baculovirus late p6.9 promoter (Hill-Perkins and Possee, 1990). Compared to the routinely used very late polyhedrin promoter, the p6.9 promoter drives expression at earlier stages of infection when cells are more likely in good condition and therefore the aggregation of expressed foreign proteins could be avoided (Ishiyama and Ikeda, 2010, Li et al., 2012, van Oers, 2011).

The 4V1 acceptor vectors (4V1G and 4V1R) carry an N-terminal HA-tagged TEV protease coding sequence followed by the TEV protease cleavage site (TCS) and a Twin-Strep tag coding sequence followed by a recognition site for enterokinase. Between multiple cloning site (MCS) 1 and 2, there is a LacZ-alpha expression cassette, which allows blue/white selection of recombinant clones. Downstream of MCS2, there is another TCS and an EGFP (4V1G) or tagRFP (4V1R) coding sequence. The fluorescent and target proteins can be expressed as a single ORF. By observing the fluorescence of infected cells, we can easily determine whether the target is expressed.

In the 5V1T acceptor vectors (5V1TG and 5V1TR), different from 4V1 acceptor vectors, the TEV protease and EGFP (5V1TG) or tagRFP (5V1TR) coding sequences are fused and expressed as a GP64 promoter-driven ORF.

The 4V2 donor vectors (4V2G and 4V2R) carry an N-terminal 10×His coding sequence followed by a recognition site for enterokinase. Both vectors contain a kanamycin resistance marker. The screening region is composed of a high-copy PUC origin of replication and a LacZ-alpha expression cassette, flanked by MCS1 and MCS2. Downstream of MCS2, there is a TCS and a fluorescent protein (EGFP in 4V2G and tagRFP in 4V2R) coding sequence. The expression of the target protein is driven by the very late p10 promoter. The 4V2 vectors also contain the conditional origin of replication, R6Kγ. Once the screening region is replaced by a foreign gene, the donor vector only contains the R6Kγ origin and can only be propagated in *E. coli* strains with the pir + genotype.

There are several single restriction sites located on both sides of the p6.9 and p10 promoter regions in the 4V1/5V1 acceptor and 4V2 donor vectors, respectively. As a result they can be replaced by other baculovirus promoters for a specific purpose.

### 2.2 Schemes for the expression of large multiprotein complexes

**Fig. 2:**
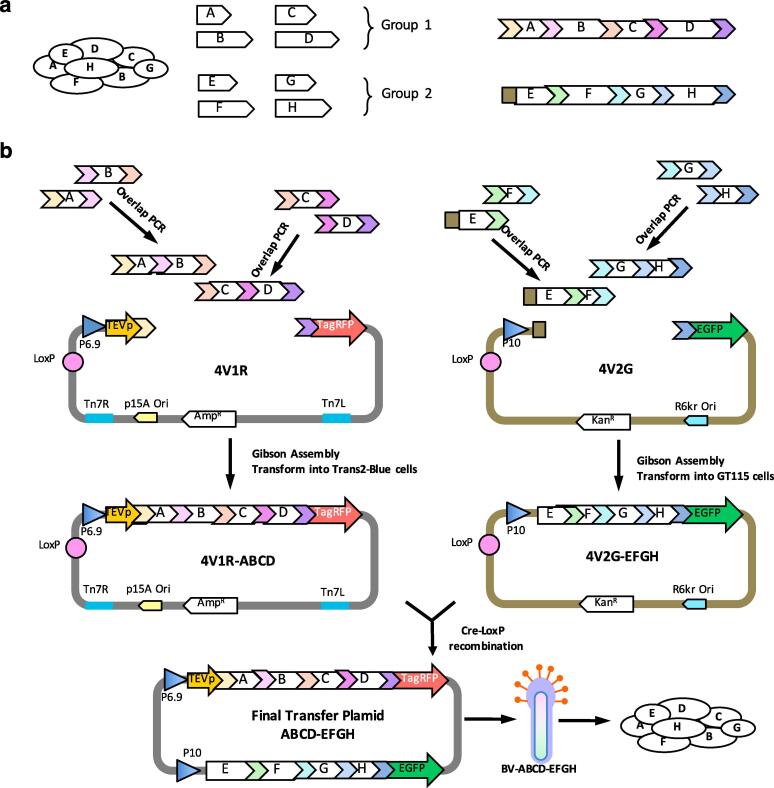

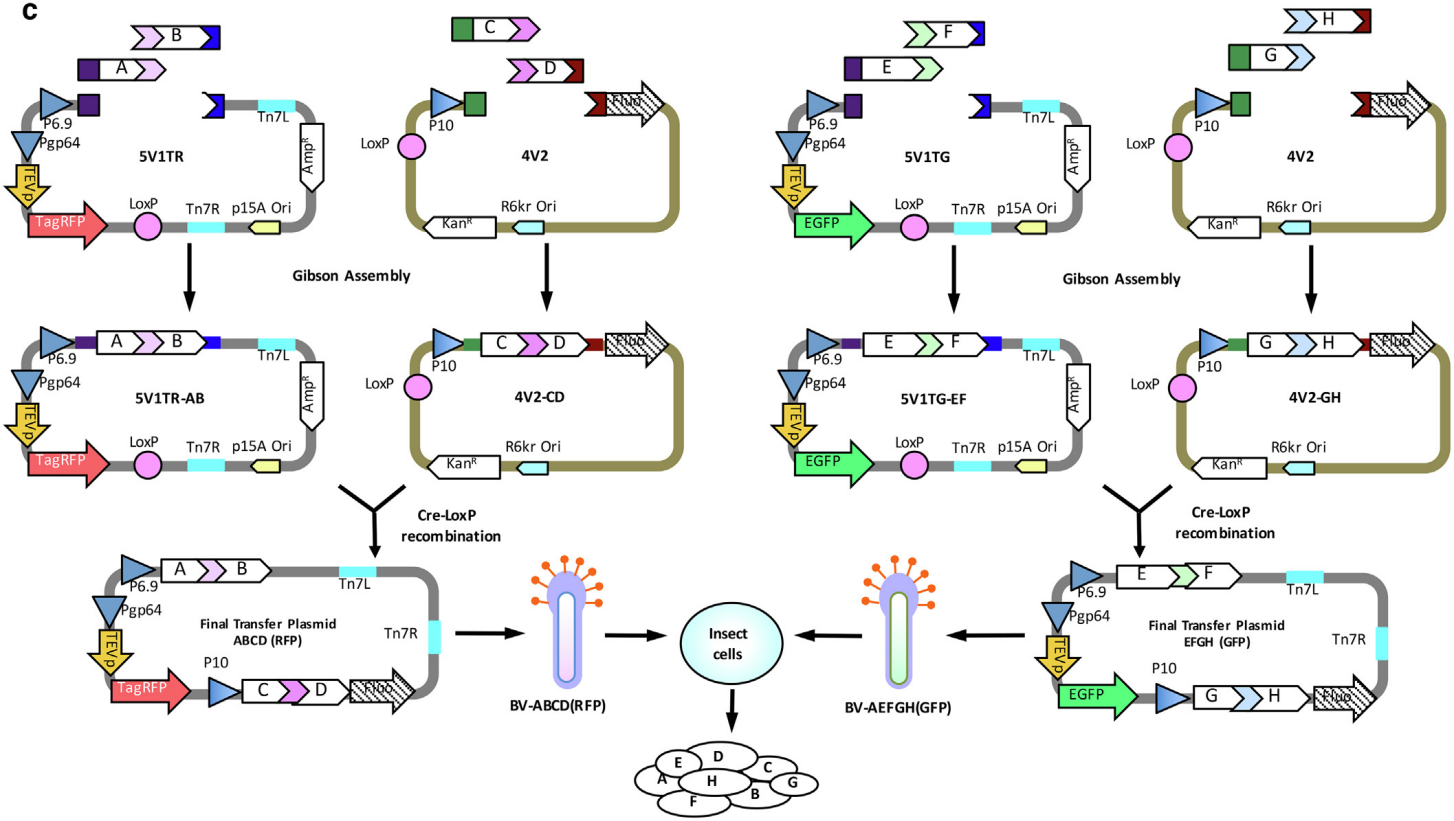
Schemes for the expression of large multiprotein complexes. (a) The eight-subunit protein complex to be expressed. The eight genes are divided into two groups according to their sizes. Two long polyproteins are designed with TEV cleavage sites separating the adjacent genes. Represents (b) Schematic representation of Scheme 1 for the expression of multiprotein complexes with a molecular weight less than 600 kDa. Here the acceptor vector 4V1R is used, but 5V1TR can also be used. (c) Schematic representation of Scheme 2 for the expression of multiprotein complexes with a molecular weight greater than 600 kDa. The fluorescent protein in the 4V2G/4V2R donor vector is not expressed because a stop codon has been inserted at the end of the fusion gene, which is located at the upstream of the coding sequence of the fluorescent protein. The coding sequences of EGFP and tagRFP can also be removed by restriction enzyme digestion.

The SmartBac system was designed for easier and faster expression of large multiprotein complexes in insect cells. A variety of experimental schemes could be applied to produce the final transfer plasmids. Here we present two schemes to use SmartBac system. In Fig. 2a, a vector is designed to express a multiprotein complex composed of eight different subunits (subunits A, B, C, D, E, F, G and H) in insect cells. If the molecular weight of the complex is less than 600 kDa, we propose using Scheme 1 (Fig. 2b). The eight subunits are divided into two groups so that the sum of the lengths of the genes in one group is as similar as possible to the other group. Then for each group of genes, a fusion DNA fragment (ABCD and EFGH) with TCS coding sequences separating the adjacent genes is designed.

Next, the long ABCD/EFGH fragments are further divided into two short DNA fragments AB/EF and CD/GH, which can be obtained easily by overlapping PCR (Fig. 2b). To avoid unnecessary trouble in overlapping PCR, the TCS described above should be coded by multiple degenerate sequences (Supplementary Materials and Methods S2). Then, fragments AB and CD are assembled together with a linearized SmartBac RFP-expressing acceptor (we use 4V1R in Fig. 2b, but 5V1TR can also be used) utilizing a Gibson assembly reaction (Gibson et al., 2009). Fragments EF and GH are also assembled with a linearized SmartBac GFP-expressing donor (4V2G) using the same method. The positive recombinants can be easily selected by blue-white screening. Finally, the acceptor 4V1R-ABCD and donor 4V2G-EFGH are recombined via Cre-LoxP site-specific recombination to generate the final transfer plasmid ABCD-EFGH. After transforming this plasmid into DH10Bac competent cells, recombinant bacmid will be obtained. This bacmid will be transfected into insect cells to produce high-titer baculovirus BV-ABCD-EFGH to express the target complex.

If the molecular weight of the complex is greater than 600 kDa, the size of the final transfer plasmid constructed using Scheme 1 (Fig. 2b) will be larger than 25 kb. It is usually not easy to build such a large plasmid without experiences. And even if the construction is successful, the expression of the complex may fail because of an intrinsic genetic instability of bacmid with a large foreign gene insertion (Pijlman et al., 2003, van Oers, 2011). Spontaneous deletion of the foreign gene insertion may occur during the amplification of P2 virus (our unpublished data). In this case, we could switch to Scheme 2 (Fig. 2c). As shown in Fig. 2c, fragments A and B are assembled with linearized 5V1TR vector, and fragments C and D are fused with linearized 4V2 vector. A stop codon has been inserted by PCR so that the fluorescent protein in 4V2G and 4V2R vectors will not be expressed. The same method is used to clone fragments E, F, G and H using 5V1TG and 4V2 vectors respectively. Next, the two different transfer plasmids ABCD and EFGH are built by Cre-LoxP recombination. These plasmids will produce two types of recombinant baculoviruses (BV-ABCD and BV-EFGH), one expressing subunits A, B, C and D and tagRFP, and the other expressing subunits E, F, G and H and EGFP. Insect cells co-infected with these two baculoviruses will express the entire protein complex with the fluorescence signals of tagRFP and EGFP to monitor the relevant expression.

**Fig. 3:**
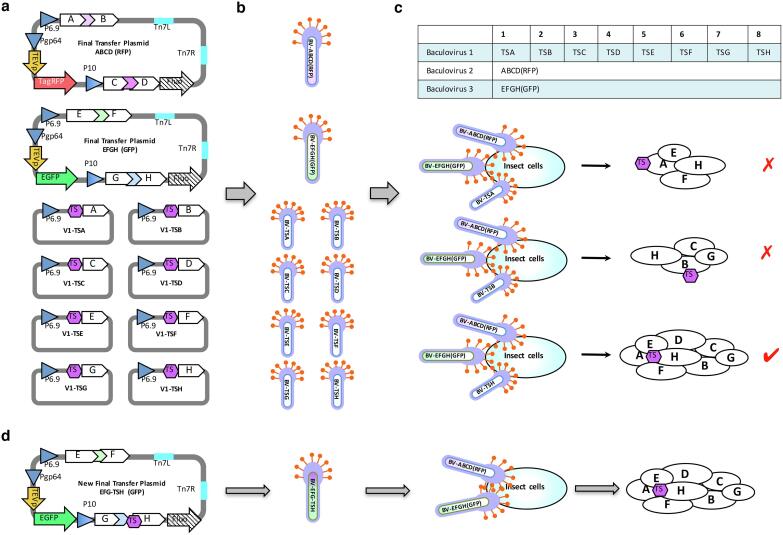
Screening for the best affinity-tagged subunit through co-infection of insect cells (Scheme 3). (a) Diagrams of the ten types of transfer plasmids. The final transfer plasmids, ABCD (RFP) and EFGH (GFP), are generated using Scheme 2, and each will express four protein subunits without affinity labels. Each of the other eight transfer plasmids will express one subunit with an N-terminal Twin-Strep (TS) tag. Either the 4V1 or 5V1 vector can be used here. (b) Production of ten types of recombinant baculoviruses (RBVs). Transformation of the ten types of plasmids into DH10Bac competent cells generates 10 types of RBVs. (c) Screening baculovirus combinations to find the subunit that results in the best purification. The ten types of RBVs are divided into eight groups, and each group contains BV-ABCD (RFP), BV-EFGH (GFP) and one BV-TSn (where n corresponds to the subunit, A to H). Insect cells are co-infected with eight groups of RBVs and strep-affinity resin is used to pull down proteins bound to the TS-tagged subunit. The tagged subunit that allows the best purification of the whole complex is selected. In this example, subunit H is the best. (d) Production of the multiprotein complex. Based on the screening result in (c), a new final transfer plasmid EFG-TSH (GFP) is constructed, which expresses an N-terminal TS-tagged subunit H. The whole protein complex will be purified from insect cells co-infected with BV-ABCD (RFP) and BV-EFG-TSH (GFP).

The SmartBac acceptors carry an optional N-terminal Twin-Step-tag sequence, and the donors carry an optional N-terminal His-Tag sequence. Either tag can be fused to a target subunit and used to purify the whole complex by affinity chromatography. If there is enough prior knowledge of the structure of the complex, it is easy to determine the most suitable subunit to fuse with an affinity tag. However, when prior knowledge is limited, screening an appropriate subunit would be important because different affinity-tagged subunits would affect the effectiveness and successfulness of purifying the entire complex. Imagine that we are expressing an eight-subunit complex and are not sure which subunit is suitable for labeling a tag. If we use a classical “Trial and Error” approach and construct multiple large final transfer plasmids, the workload would be extremely high. To solve this issue, we propose a simple and universal scheme using SmartBac (Scheme 3, see Fig. 3). Two large final transfer plasmids ABCD and EFGH are built according to Scheme 2 (Fig. 2c) but where none of the eight subunits are labeled with affinity tags (Fig. 3a). An additional eight smaller transfer plasmids (from V1-TSA to V1-TSH) based on one acceptor (4V1G, 4V1R, 5V1TG or 5V1TR) are constructed, each containing one N-terminal Twin-Strep-tagged subunit. A total of ten recombinant baculoviruses are obtained, including BV-ABCD, BV-EFGH and BV-TSn (where n ranges from A to H) (Fig. 3b). The insect cells are co-infected with three baculoviruses, BV-ABCD, BV-EFGH and one type of BV-TSn. The baculovirus combinations used for screening are shown in Fig. 3c. After purification, we know affinity-tagged subunit H results in the best efficient purification of the entire complex. To increase yield and obtain a more homogenous sample, a new intermediate vector (EFG-TSH) containing tagged subunit H is built (Fig. 3d). The resulting new recombinant baculovirus, BV-EFG-TSH, together with the existing recombinant baculovirus, BV-ABCD, are eventually used to express the entire complex. And of course the efficiency of infection and expression can also be monitored in real time by observing the fluorescence signal of co-expressed EGFP and tagRFP.

### 2.3 Multiprotein complexes expressed using SmartBac system

**Fig. 4:**
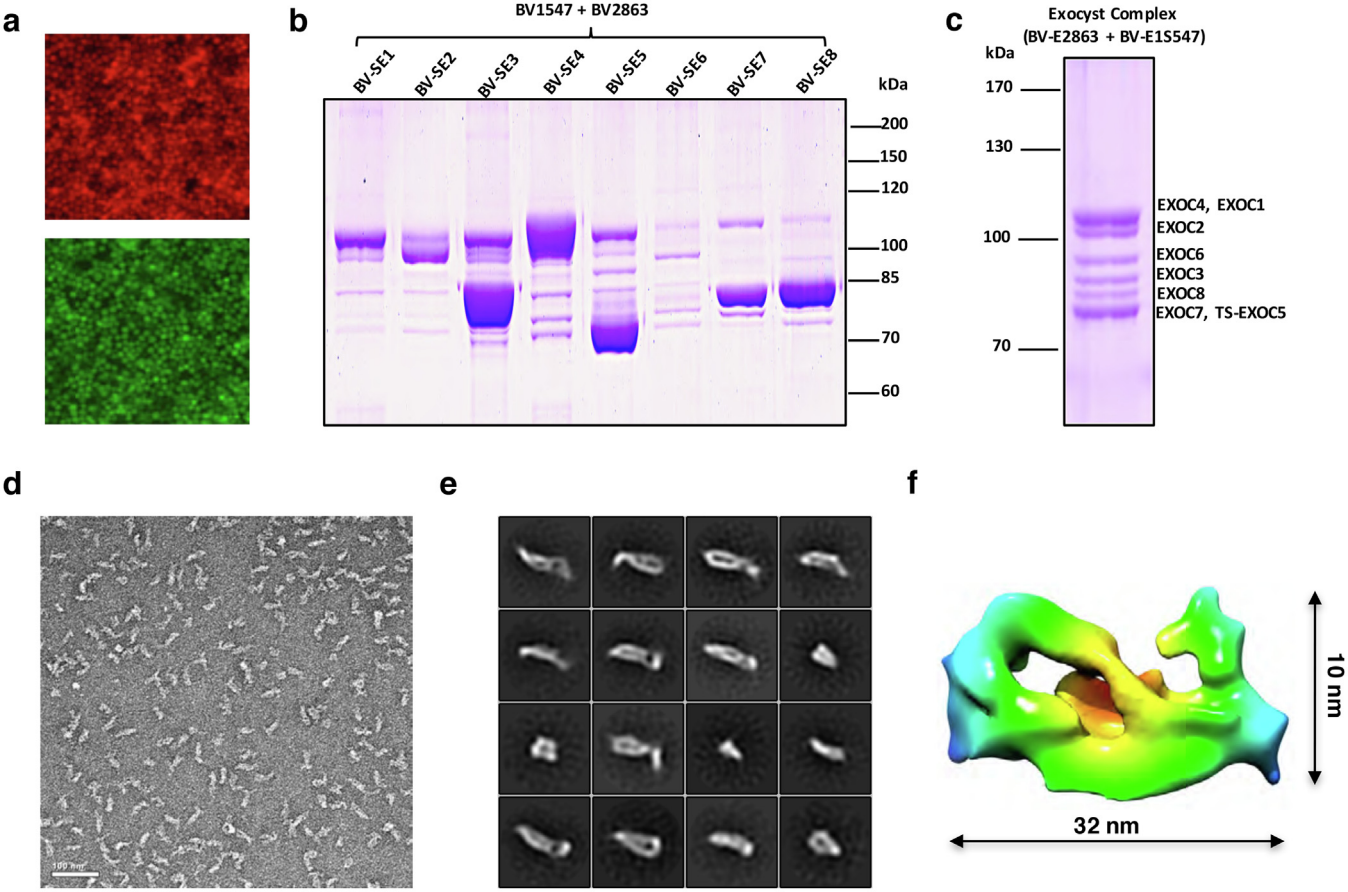

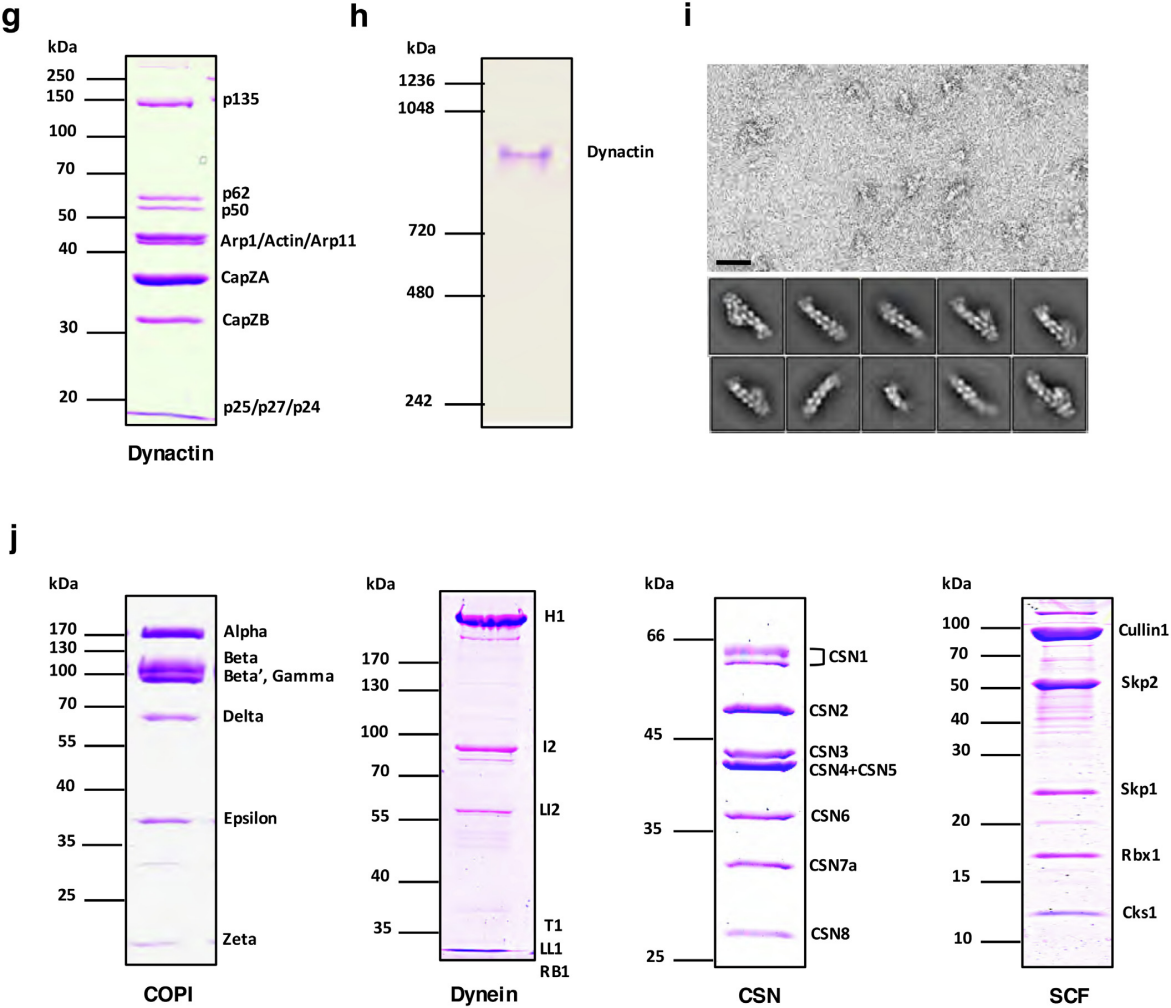
Examples of multiprotein complexes expressed using the SmartBac system. (a) Fluorescence signals for tagRFP (top) and EGFP (bottom) detected from Sf9 cells transfected with BVE1S547 and BV2863 (see Table 1). (b) Coomassie-stained SDS-PAGE gel of human exocyst complex purified using eight different Twin-Strep tagged subunits (BV-SE1 to BV-SE8, see Table 1). (c) Coomassie-stained SDS-PAGE gel of human exocyst complex purified from insect cells co-infected with BV-2863 and BV-E1S547 (see Table 1). The exocyst complex was purified using Twin-Strep-tagged subunit EXOC5. (d) Electron micrograph of negative-stained recombinant human exocyst complex. The bar represents 100 nm. (e) Representative classes from 2D classification of recombinant human exocyst complex particles. (f) 3D reconstruction of recombinant human exocyst complex based nsEM data. (g) Coomassie-stained SDS-PAGE gel of the human dynactin complex purified by one-step strep-affinity purification. (h) Coomassie-stained 3–8% Native-PAGE gel of purified human dynactin complex after glycerol density gradient centrifugation purification. (i) Single-particle nsEM analysis of recombinant human dynactin complex with the representative raw micrograph (top) and 2D class averages (bottom). Scale bar, 50 nm. (j) Coomassie-stained SDS-PAGE gel of purified recombinant human COPI complex, human dynein complex, human CSN complex and human SCF complex.

To test the SmartBac system, we expressed the human exocyst complex in insect cells. The exocyst complex is responsible for tethering secretory vesicles to the plasma membrane in preparation for soluble *N*-ethylmaleimide-sensitive factor (NSF) attachment protein receptor (SNARE) mediated membrane fusion (Wu and Guo, 2015). The human exocyst complex contains eight evolutionary conserved subunits—EXOC1 (102 kDa), EXOC2 (104 kDa), EXOC3 (86 kDa), EXOC4 (110 kDa), EXOC5 (82 kDa), EXOC6 (94 kDa), EXOC7 (78 kDa) and EXOC8 (82 kDa). Because the published literature does not provide information about which subunit is the most suitable for complex purification, we used Scheme 3 (Fig. 3) to screen the target subunits. All of the vectors we built for exocyst expression are shown in Table 1 (see also Supplementary Materials and Methods S3). First, we constructed two types of recombinant baculoviruses, BV-E1547 (EXOC1, 5, 4, 7) and BV-E2863 (EXOC2, 8, 6, 3), to express the eight subunits without any tags according to Scheme 2 (Fig. 2c). These baculoviruses also expressed tagRFP and EGFP, respectively, which allowed us to conveniently determine whether virus infection and protein expression were successful (Fig. 4a). We also produced eight additional types of recombinant baculoviruses, each expressing an individual subunit with an N-terminal Twin-Strep tag (BV-SE1 to BV-SE8). Then we co-infected insect cells with BV-E1547, BV-E2863 and a baculovirus expressing a single affinity tagged-subunit (BV-SE1 to BV-SE8). The best purification of the entire exocyst complex was achieved using BV-SE5 (Fig. 4b). Then we constructed a new donor vector 4V2-E1S5 that contains EXOC1 and N-terminal Twin Strep-tagged EXOC5. Recombination between 4V2-E1S5 and 5V1TR-E47 (containing EXOC4 and EXOC7) produced a new final transfer plasmid E1S547 (Table 1), from which recombinant baculovirus BV-E1S547 was obtained. Insect cells were co-infected with BV-E1S547 and BV-2863. After one-step strep-affinity purification, the entire exocyst complex with high purity was obtained (Fig. 4c). The tethering activity of this purified exocyst complex was determined via *in vitro* liposome tethering assay (to be published elsewhere). Negative-staining electron microscopy (nsEM) of the sample showed homogenous rod-like particles (Fig. 4d). Preliminary 2D classification of nsEM images (Fig. 4e) and 3D reconstruction (Fig. 4f) indicate that the human exocyst complex exhibits a similar dimension and shape with the extracted exocyst complex from yeast (Heider et al., 2016, Mei et al., 2018). The detailed information about primer design, molecular cloning, cell transfection, protein expression and purification, and electron microscopy is described in Materials and Methods.

**Table 1:** Recombination of human Exocyst complex using SmartBac system.

Subunit	Intermediate plasmid	Final transfer plasmid	Recombinant baculovirus

TS-tagged EXOCn		5V1TG-SEn	BV-SEn
EXOC2, EXOC8	4V2-E28	E2863	BV-E2863
EXOC6, EXOC3	5V1TG-E63		
EXOC1, EXOC5	4V2-E15	E1547	BV-E1547
EXOC4, EXOC7	5V1TR-E47		
EXOC1, TS-tagged EXOC5	4V2-E1SE5	E1S547	BV-E1S547

We also reconstituted the human dynactin complex using the SmartBac system. Dynactin is a multiprotein complex that works with cytoplasmic dynein to transport cargo along microtubules. It is a large complex of approximately 1.2 MDa composed of 23 subunits corresponding to 11 different types of proteins (Reck-Peterson, 2015). Dynactin is built around a short actin-like filament composed of Arp1 (43 kDa, 8 copies) and β-actin (42 kDa, 1 copy). The barbed end and the pointed end of this filament are capped by the CapZα-CapZβ complex (33 kDa, 31 kDa) and the Arp11-p25-p27-p62 complex (46 kDa, 20 kDa, 21 kDa, and 52 kDa) respectively. The shoulder complex, which is made up of p50 (45 kDa, 4 copies), p24 (21 kDa, 2 copies) and p150/p135 (142 kDa/127 kDa, 2 copies), is positioned toward the barbed end of the Arp1 filament (Urnavicius et al., 2015). As shown in Table 2, three types of vectors were used to express the 11 subunits of the dynactin complex, and the N-terminal Twin-Strep tag on p135 was used to purify the whole complex. The shoulder complex proteins, p135, p50 and p24, were expressed by BV-M5, which was generated from the plasmid 5V1TG-M5. The final transfer plasmid AB was obtained through recombination of the acceptor 5V1TR-B and donor 4V2-A. Plasmid AB was then used to produce the recombinant baculovirus BV-AB expressing the other eight dynactin subunits. Insect cells were co-infected with BV-M5 and BV-AB to express the entire dynactin complex. After one-step strep-affinity purification, the dynactin complex was purified well with rational stoichiometry of its subunits (Fig. 4g). After glycerol density gradient ultracentrifugation, the further purified dynactin complex exhibited a single visible band on a native gel (Fig. 4h), suggesting a high homogeneity of the specimen. This recombinant human dynactin complex sample was further investigated by nsEM and subsequent 2D class classification (Fig. 4i), showing a rod-like particle with a shoulder at one end, which is consistent with the endogenous dynactin complex purified from pig brains (Urnavicius et al., 2015).

**Table 2:** Recombination of human Dynactin complex using SmartBac system.

Subunit	Intermediate plasmid	Final transfer plasmid	Recombinant baculovirus

TS-tagged p135	5V1TG-M5	5V1TG-M5	BV-M5
p50			
p24			
Arp1	4V2-A	AB	BV-AB
Beta-actin			
CapZ alpha	5V1TR-B		
CapZ beta			
P25(DCTN5)			
P27(DCTN6)			
Arp11			
P62(DCTN4)			

Besides the human exocyst and dynactin complexes, we also successfully expressed many other protein complexes using the SmartBac system (Fig. 4j). These include the human COPI complex (7 subunits, 558 kDa) (Wang et al., 2016), cytoplasmic Dynein complex (12 subunits, 1380 kDa) (Zhang et al., 2017), CSN complex (8 subunits, 343 kDa) (Mosadeghi et al., 2016) and SCF complex (5 subunits, 180 kDa) (Zheng et al., 2002). The recombinant COPI complex sample has been used to study the structure of coatomer in its soluble form (Wang et al., 2016). These results indicate that our SmartBac system can be used to efficiently express a wide range of large multiprotein complexes.

## 3 Discussions

Obtaining large multiprotein complexes through recombinant expression has always been challenging for researchers who need a sufficient quantity of high-purity sample for structural and biochemical studies. The key to successful protein production using the baculovirus expression system is the construction of the final transfer plasmid containing genes of multiple protein subunits. The classical MultiBac system uses polycistronic vectors carrying multiple GECs for the expression of multiprotein complexes (Berger et al., 2004). This expression strategy requires first constructing the junior plasmids, each containing only one GEC. Then several rounds of plasmid construction are needed to obtain multi-GEC donor and acceptor vectors. The final transfer plasmid carrying all GECs is produced by Cre-LoxP recombination. MultiBac has been proven powerful in generating multiprotein complexes (Berger et al., 2013, Vijayachandran et al., 2011), especially when robotic support is available. But in ordinary laboratories without robotics, the first two procedures require a lot of labor and time. And as more GECs are added to a single vector, the difficulty of plasmid construction increases, which is due to the increasing size of the plasmid and the lack of an efficient screening method for large positive recombinants.

The biGBac system developed in recent years has more advantages in assembling multiple GECs (Weissmann and Peters, 2018, Weissmann et al., 2016). This system uses computationally optimized DNA linker for efficient Gibson assembly reactions to assemble multiple GECs. Although the junior plasmids pLIBs, each containing only one GEC, still need to be constructed first, the final transfer plasmid containing multiple GECs can be assembled by two rounds of Gibson assembly reactions. In the first round, the pBIG1 vectors each containing up to 5 GECs are produced. Then the pBIG2 vectors containing more GECs can be assembled from pBIG1 vectors during the second round. The biGBac system does not depend on combining “donor” and “acceptor” vectors; as a result any biGBac construct can be used for the generation of baculoviruses. This system has been successfully applied to express the human anaphase promoting complex APC/C that contains 17 different subunits with a total molecular weight of 1.2 MDa (Weissmann et al., 2016). However, the efficiency of Gibson assembly would decrease when the number and length of assembled DNA fragments increase. Even using the computationally optimized DNA linker, the averaged efficiency of producing pBIG1 constructs from the pLIBs was less than 22% (42/196) (Weissmann et al., 2016). In addition, because all cDNAs are cloned into pLIB vectors, all GECs contain the same promoter and terminator. This would produce a large accumulation of repeating sequences in the final pBIG2 vector. Whether these repeating sequences would increase the possibility of unpredicted recombination and gene loss should be presumably considered.

In the present work, we developed a new baculovirus expression system SmartBac. We mimicked the polyprotein production strategy to realize the expression of multiple subunits (Table 3). Although this strategy has already been discussed and applied (Bieniossek et al., 2012, Chen et al., 2010, Nie et al., 2014), a specialized vector system and standardized procedures are not available. In SmartBac system, long DNA sequences encoding polyproteins are produced by overlapping PCR and Gibson assembly. There is no need to construct junior vectors in comparison with MultiBac and biGBac (Table 3). To ensure high assembly efficiency, we usually use three DNA fragments for Gibson reactions. The acceptor and donor vectors support blue-white screening, which increases the selection efficiency of positive clones. The final transfer plasmid produced by Cre-LoxP recombination is pre-designed to a size of less than 25 kb, which allows an efficient chemical transformation in the subsequent procedure. To increase the stability of the large final transfer plasmid propagated in *E. coli*, we introduced a low-copy p15A replication origin into the acceptor vectors and cultured the bacteria at 30 °C.

**Table 3:** Comparisons among MultiBac, biGBac and SmartBac systems.

		MultiBac	biGBac	SmartBac
Expression Strategy		Multi-GEC	Multi-GEC	Polyprotein & Multi-GEC
Vectors		Acceptors (pACEBac1, pACEBac2) Donors (pIDS, pIDK, pIDS)	pLIB, pBIG1, pBIG2	Acceptors (4V1G, 4V1R, 5V1TG, 5V1TR) Donors (4V2G, 4V2R)
Vectors that can produce recombinant baculoviruses		Only acceptors	pLIB, pBIG1, pBIG2	Only acceptors
Molecular cloning	Construction of junior plasmid containing one GEC	Use the acceptors and donors	Use only the pLIB	This step is not needed.
	Construction of vectors containing multiple genes	Construct large acceptors and large donors using the multiplication module consisting of homing endonuclease and BstXI	Construct pBIG1 vectors by linker-optimized Gibson assembly	Construct large acceptors and large donors by overlapping PCR and Gibson assembly
	Method to screen positive clones	None	None	Blue-White screening
	Construction of vectors containing more genes	Recombine large acceptor and large donor by Cre-LoxP combination	Construct pBIG2 vector by linker-optimized Gibson assembly from pBIG1 vectors	Recombine large acceptor and large donor by Cre-LoxP combination
Whether the vector supports expression monitoring		No	No	Yes (using 4V1G and 4V1R)
Whether the vector supports transfection and virus amplification monitoring		No	No	Yes
Whether the vector supports monitoring co-infection of two kinds of viruses		No	No	Yes
Reported largest protein complexes expressed		Dynein complex (6 cDNAs, 1.4 MDa)	APC/C related complex (17 cDNAs, 17 subunits, 1.2MDa)	Dynactin complex (11 cDNAs, 23 subunits, 1.2MDa)

The polyprotein strategy is useful in case of that the assembly of the complex is sensitive to subunit stoichiometry. In our experiment, we found the polyprotein strategy would yield a more homogenous expression of human COPI complex (Wang et al., 2016). In other cases, the subunits may have different copies. For example, in human dynactin complex, the copy number ratio of subunits p135 (or p150):p50:p24 is 2:4:2. To ensure a correct subunit stoichiometry, we designed a polyprotein expressing 1 copy of p135, 2 copies of p50 and 1 copy of p24. If the number of one subunit is much higher than the other subunits, we will use a stronger promoter (e.g. the polyhedrin promoter) to express this subunit independently.

The design of polyprotein is very important. In our experience, besides the length of the final DNA, the order of subunits in the polyprotein should also be considered. This order will affect the amino acid sequences of the C-terminus or N-terminus of these subunits. The polyprotein strategy puts a TEV recognition site (ENLYFQS) between adjacent subunits. In vivo TEV processing of the polyprotein leads to the production of mature subunits, which will yield one additional serine at the N-terminus and/or a six amino acid peptide ENLYFQ at the C-terminus for each subunit. If the N-terminus or the C-terminus of one subunit is vital to the assembly of the final complex, these extra amino acids would adversely affect the success of the complex assembly. Under these circumstances, the location of this specific subunit in the polyprotein should be considered carefully. Expressing this subunit in an individual GEC is another good option. In human dynactin complex, the C-terminus of Arp1 is very important to the assembly of actin-like filament, thus we expressed it using a single GEC so that no extra amino acids exist in its C-terminus.

In comparison with MultiBac and biGBac (Table 3), we explicitly introduced EGFP and tagRFP genes into the SmartBac system, which allows efficient and direct real-time monitoring viral infection and protein expression. In 4V1 vectors, the TEV protease and the fluorescent proteins are co-expressed with the designed polyprotein within one ORF. We also designed 5V1 vectors that use a different GEC to express the fluorescent protein and TEV protease to avoid potential insufficient cleavage of the fluorescent protein.

Besides, we also provided a standard procedure using SmartBac system to screen an optimal tagged subunit for an efficient purification of the target complex. The successful expression and purification of six large multiprotein complexes including the human exocyst complex and dynactin complex have proven the wide applicable potential of SmartBac system.

Overall, with the wide application of cryo-EM, more and more research groups are carrying out structural and functional studies of multiprotein complexes. In addition to MultiBac and biGBac (Table 3), SmartBac system provides another good baculovirus expression option to get recombinant expression of large multiprotein complexes with sufficient quantity for the subsequent structural and biochemical studies.

## 4 Materials and methods

### 4.1 Vector construction

A portion of the 4V1 vectors was derived from pFastbacDUAL (Invitrogen, USA). 4V2 vectors and all other portions of the 4V1 vectors were synthesized by Genewiz, China. The DNA fragments were fused together via Gibson assembly (E2611, NEB, England) to generate the 4V1G, 4V1R, 4V2G and 4V2R vectors. Then, using 4V1 vectors, 5V1 vectors were generated by Gibson assembly and other classical molecular cloning methods. The sequences of the SmartBac vectors are shown in Supplementary Materials and Methods S1. We recommend using SnapGene Viewer (http://www.snapgene.com/) to view the plasmid maps.

### 4.2 Gibson assembly reactions

The linearized plasmid fragments were obtained by restriction enzyme digestion or PCR using Q5 High-fidelity DNA Polymerase (M0492, NEB, England). DNA fragments to be inserted into the SmartBac vectors were amplified by PCR to produce the appropriate overlaps. The overlapping primers were designed according to NEB instruction manual for E2611. Assembly was done in a 15–20 μl reaction volume with 0.2–0.3 pmols each DNA fragment. Samples were incubated in a thermocycler at 50 °C for 60 min.

### 4.3 Blue-white selection of positive recombinants

To perform blue-white selection, 2.5 μl assembled product was added to 100 μl chemically competent cells. The 4V1- and 5V1-based constructs were transformed into chemically competent Mach1™-T1R (Invitrogen) or DH5alpha or Trans2-blue (TransGen Biotech, China) cells. After 1 h recovery in SOC medium at 37 °C, cells were plated onto LB agar plates containing 100 μg/ml ampicillin, 40 μg/ml IPTG and 100 μg/ml Bluo-gal. The 4V2-based constructs were transformed into chemically competent GT115 cells (InvivoGen, USA), and then cells were plated onto LB agar plates containing 50 μg/ml kanamycin, 40 μg/ml IPTG and 100 μg/ml Bluo-gal. Single white colonies were picked and grown in 5 ml LB medium with the proper antibiotics for further plasmid extraction and PCR analysis. The positive recombinants were sequenced at BioSune, China.

### 4.4 Production of the final transfer plasmid by Cre-LoxP recombination

The donor and acceptor vectors (0.1 pmols each) were mixed with 1 μl Cre recombinase (M0298, NEB) in a 20 μl reaction and incubated at 30 °C for 1 h. Ten microliters of the reaction mixture were added to 100 μl chemically competent Trans2-blue cells. After heat-shock at 42 °C for 30 s, 500 μl SOC medium was added, and the suspension was incubated at 37 °C for 1 h with shaking (if the size of the recombined vector was larger than 15 kb, the suspension was incubated at 30 °C for 4 h). The cell suspension was plated on LB agar plates containing 50 μg/ml kanamycin and 100 μg/ml ampicillin. The plates were incubated at 37 °C overnight (or 30 °C for 24 h). Positive colonies were verified by PCR using the primers Loxp-F (5′-CCACTGCGCCGTTACCAC-3′) and Loxp-R (5′-GCCGGTATGTACAGGAAG-3′). A 375 bp PCR product was amplified from positive clones. The final transfer plasmids were extracted from the positive clones.

### 4.5 Production of recombinant baculovirus

Chemically competent DH10Bac cells were transformed with the final transfer plasmid according to the Bac to Bac manual instructions (Invitrogen). For transformation of large plasmids, the transformation mixture was incubated at 30 °C with shaking for 8–12 h and plates were incubated at 30 °C for more than 48 h. Single white colonies were inoculated into 5 ml LB medium containing 50 μg/ml kanamycin, 14 μg/ml gentamicin, and 10 μg/ml tetracycline. Recombinant bacmids were extracted and verified by PCR amplification with three pairs of primers (Tn7R: 5′-GTTTTCCCAGTCACGAC-3′ and 5′-AAGTTTGAGCAGCCGCGTAG-3′; Tn7L: 5′-CAGGAAACAGCTATGAC-3′ and 5′-ACCTCCCCCTGAACCTGAAA-3′; Empty: 5′-GTTTTCCCAGTCACGAC-3′ and M13 Reverse: 5′-CAGGAAACAGCTATGAC-3′). Using the “Tn7R” and “Tn7L” primer pairs, PCR products of 661 bp and 521 bp, respectively, are amplified from recombinant bacmids. If the recombinant bacmid is contaminated with wild-type bacmid, a PCR product of 300 bp will produce using the “Empty” primer pairs. It is recommended to verify the existence of all of the subunit genes in the recombinant bacmids by PCR if the size of final transfer plasmid is larger than 20 kb.

### 4.6 Transfection and virus production in insect cells

Transfection and Baculovirus production were done according to the Bac to Bac manual (Invitrogen, USA). Successful transfection was determined by the expression of EGFP and/or tagRFP fluorescent proteins. P2 virus was used for expression.

### 4.7 Construction, expression and purification of the human exocyst complex

All human exocyst genes were purchased from Origene, USA. For each gene, two cycles of PCR were done to produce the target overlapped ends (M0492, NEB). 4V2G was digested by NdeI, KpnI and EcoRI (NEB). The 2.5 kb linearized vector fragment 4V2 was recovered and assembled with the PCR products of EXOC1 (using E1F1 and E1R1, E1F2 and E1R2 primers) and EXOC5 (using E5F1 and E5R1, E5F2 and E5R2 primers), and EXOC2 (using E2F1 and E2R1, E2F2 and E2R2 primers) and EXOC8 (using E8F1 and E8R1, E8F2 and E8R2 primers), respectively (E2611, NEB) to generate 4V2-E15 and 4V2-E28. 5V1TG and 5V1TR were digested by NdeI and XhoI. Linearized 5V1TG was assembled with the PCR products of EXOC6 (using E6F1 and E6R1, E6F2 and E6R2 primers) and EXOC3 (using E3F1 and E3R1, E3F2 and E3R2 primers) to generate 5V1TG-E63. Linearized 5V1TR was assembled with the PCR products of EXOC4 (using E4F1 and E4R1, E4F2 and E4R2 primers) and EXOC7 (using E7F1 and E7R1, E7F2 and E7R2 primers) to generate 5V1TR-E47. Every EXOC gene was cloned into 5V1TG to produce 5V1TG-SEn (using EnF1 and EnR1 primers, where n refers to the subunit number from 1 to 8). To build 4V2-E1SE5, Twin-strep tagged EXOC5 coding sequence (which was obtained from 5V1TG-SE5 by PCR, using S5-F and S5-R primers) was assembled with the EXOC1 gene PCR product and linearized 4V2 vector. The transformation and identification of positive clones, and the Cre-recombination between donor and acceptor vectors were done using the same method described in Material and Methods.

Bacmid was purified according to the Bac to Bac manual (Invitrogen). Sf9 cells were cultured in ESF921 medium (Expression Systems, USA) and transfected with the appropriate bacmid using Cellfectin II (Invitrogen) in 35 mm plates. After four to seven days incubation at 28 °C the transfection efficiency was evaluated by observing EGFP and tagRFP expression using a Nikon T100 fluorescence microscope. P1 virus was collected and stored at 4 °C. A total of 200 μl P1 was added to 180 ml Sf9 cell suspension with a density of 2 × 10^6^ cells/ml, and the sample was incubated in an incubator shaker (Infors, Switzerland) at 28 °C/124 rpm.

P2 virus was collected and stored at 4 °C. For protein expression, two types of P2 baculoviruses (5 ml each) were added to 500 ml Sf9 cell suspension (at 2 × 10^6^ cells/ml), and the infected cells were incubated at 27 °C/124 rpm for 72 h. The cells were harvested by centrifugation at 2000*g* for 10 min at 4 °C (JLA 10 rotor in an Avanti J26-XP centrifuge, Beckman, USA). A frozen pellet from 2L of insect cell culture was thawed on ice and resuspended in lysis buffer (50 mM HEPES pH 8.0, 150 mM NaCl, 10% (v/v) glycerol, 1 mM DTT) supplemented with protease inhibitors (Complete-EDTA Free, Roche Applied Science, Switzerland). Cells were lysed in a 40-ml dounce-type tissue grinder (Wheaton, USA) using 30 strokes. The lysate was cleared by centrifugation (18,000 rpm, 40 min, 4 °C; JA25.5 Rotor, Beckman Coulter) and added to 1 ml pre-washed Strep-Tactin (IBA, Germany) in a 2.5 × 10 cm Econo-Column (Bio-Rad, USA). Beads were washed with 10 ml lysis buffer, and exocyst complex was eluted with lysis buffer supplemented with 10 mM desthiobiotin.

The purified exocyst complex was diluted to 0.02 mg/ml and absorbed to glow discharged GiG322 copper grids coated with thin carbon film (LifeTrust, China) for 1 min. Then the grids was washed twice by lysis buffer and stained with 4% (w/v) uranyl acetate for 2 min. Micrographs were collected on a FEI Talos F200C electron microscope (ThermoFisher, USA) operated at 200 kV. Images were collected with a 4 K × 4 K DE20 camera (Direct Electron, USA) at a nominal 28,000× magnification with a pixel size of 1.582 Å. The defocus value was range from −2.5 to −3.5 μm. Contrast transfer function (CTF) estimation was performed with Gctf (Zhang, 2016). The micrographs were then phase flipped. We semi-automatically picked particles with Gautomatch (http://www.mrc-lmb.cam.ac.uk/kzhang/Gautomatch/) and RELION (Scheres, 2012). There were 379 micrographs and 18,669 particles were selected. 2D classification were performed using RELION (Scheres, 2012). The initial model were generated by EMAN2 (Tang et al., 2007). The 3D Auto-refinement with particles bined to a pixel size of 3.172 Å. was performed using RELION (Scheres, 2012).

## 5 Competing interests

Parts of this study (SmartBac system) has been submitted to apply Chinese invention patents with the application numbers of 201610248592.8 and 201810028508.0.

## 6 Authors’ contributions

Fei S. initiated and supervised the project. YZ designed all the SmartBac systems including vectors and application strategies. YZ performed all the experiments of molecular cloning and expression constructs production. YZ, DZ, LY and Fang S. performed protein complex purification and preliminary electron microscopic characterization. YZ and Fei S. wrote the manuscript.

## References

[b0005] Bartlam M., Yang H., Rao Z. (2005). Structural insights into SARS coronavirus proteins. Curr. Opin. Struct. Biol..

[b0010] Belyaev A.S., Roy P. (1993). Development of baculovirus triple and quadruple expression vectors: co-expression of three or four bluetongue virus proteins and the synthesis of bluetongue virus-like particles in insect cells. Nucl. Acids Res..

[b0015] Berger I., Fitzgerald D.J., Richmond T.J. (2004). Baculovirus expression system for heterologous multiprotein complexes. Nat. Biotechnol..

[b0020] Berger I., Garzoni F., Chaillet M., Haffke M., Gupta K., Aubert A. (2013). The multiBac protein complex production platform at the. EMBL J. Vis. Exp..

[b0025] Bieniossek C., Imasaki T., Takagi Y., Berger I. (2012). MultiBac: expanding the research toolbox for multiprotein complexes. Trends Biochem. Sci..

[b0030] Birnbaum M.E., Berry R., Hsiao Y.S., Chen Z., Shingu-Vazquez M.A., Yu X., Waghray D., Fischer S., McCluskey J., Rossjohn J., Walz T., Garcia K.C. (2014). Molecular architecture of the alphabeta T cell receptor-CD3 complex. Proc. Natl. Acad. Sci. U.S.A..

[b0170] Carter M., Shieh J.C. (2010). Chapter 9 - Molecular cloning and recombinant DNA technology. Guide to Research Techniques in Neuroscience.

[b0035] Chang L., Zhang Z., Yang J., McLaughlin S.H., Barford D. (2015). Atomic structure of the APC/C and its mechanism of protein ubiquitination. Nature.

[b0040] Chen X., Pham E., Truong K. (2010). TEV protease-facilitated stoichiometric delivery of multiple genes using a single expression vector. Protein Sci..

[b0045] Chung Y.C., Huang J.H., Lai C.W., Sheng H.C., Shih S.R., Ho M.S., Hu Y.C. (2006). Expression, purification and characterization of enterovirus-71 virus-like particles. World J. Gastroenterol..

[b0050] des Georges A., Clarke O.B., Zalk R., Yuan Q., Condon K.J., Grassucci R.A., Hendrickson W.A., Marks A.R., Frank J. (2016). Structural basis for gating and activation of RyR1. Cell.

[b0055] Filutowicz M., McEachern M., Greener A., Mukhopadhyay P., Uhlenhopp E., Durland R., Helinski D. (1985). Role of the pi initiation protein and direct nucleotide sequence repeats in the regulation of plasmid R6K replication. Basic Life Sci..

[b0060] Gibson D.G., Young L., Chuang R.Y., Venter J.C., Hutchison C.A., Smith H.O. (2009). Enzymatic assembly of DNA molecules up to several hundred kilobases. Nat. Methods.

[b0065] Gu J., Wu M., Guo R., Yan K., Lei J., Gao N., Yang M. (2016). The architecture of the mammalian respirasome. Nature.

[b0070] Heider M.R., Gu M., Duffy C.M., Mirza A.M., Marcotte L.L., Walls A.C., Farrall N., Hakhverdyan Z., Field M.C., Rout M.P., Frost A., Munson M. (2016). Subunit connectivity, assembly determinants and architecture of the yeast exocyst complex. Nat. Struct. Mol. Biol..

[b0075] Hill-Perkins M.S., Possee R.D. (1990). A baculovirus expression vector derived from the basic protein promoter of Autographa californica nuclear polyhedrosis virus. J. Gen. Virol..

[b0080] Hitchman R.B., Possee R.D., King L.A. (2012). High-throughput baculovirus expression in insect cells. Methods Mol. Biol..

[b0085] Hu Y.C., Hsu J.T., Huang J.H., Ho M.S., Ho Y.C. (2003). Formation of enterovirus-like particle aggregates by recombinant baculoviruses co-expressing P1 and 3CD in insect cells. Biotechnol. Lett..

[b0090] Ishiyama S., Ikeda M. (2010). High-level expression and improved folding of proteins by using the vp39 late promoter enhanced with homologous DNA regions. Biotechnol. Lett..

[b0095] Jarvis D.L. (2009). Baculovirus-insect cell expression systems. Methods Enzymol..

[b0100] Kee Y., Yang K., Cohn M.A., Haas W., Gygi S.P., D'Andrea A.D. (2010). WDR20 regulates activity of the USP12 × UAF1 deubiquitinating enzyme complex. J. Biol. Chem..

[b0105] Letts J.A., Fiedorczuk K., Sazanov L.A. (2016). The architecture of respiratory supercomplexes. Nature.

[b0110] Li S.F., Wang H.L., Hu Z.H., Deng F. (2012). Genetic modification of baculovirus expression vectors. Virol. Sin..

[b0115] Liu Q., Li M.Z., Leibham D., Cortez D., Elledge S.J. (1998). The univector plasmid-fusion system, a method for rapid construction of recombinant DNA without restriction enzymes. Curr. Biol..

[b0120] Luckow V.A., Lee S.C., Barry G.F., Olins P.O. (1993). Efficient generation of infectious recombinant baculoviruses by site-specific transposon-mediated insertion of foreign genes into a baculovirus genome propagated in Escherichia coli. J. Virol..

[b0125] Mei K., Li Y., Wang S., Shao G., Wang J., Ding Y., Luo G., Yue P., Liu J.J., Wang X., Dong M.Q., Wang H.W., Guo W. (2018). Cryo-EM structure of the exocyst complex. Nat. Struct. Mol. Biol..

[b0130] Metcalf W.W., Jiang W., Wanner B.L. (1994). Use of the rep technique for allele replacement to construct new Escherichia coli hosts for maintenance of R6K gamma origin plasmids at different copy numbers. Gene.

[b0135] Mosadeghi R., Reichermeier K.M., Winkler M., Schreiber A., Reitsma J.M., Zhang Y., Stengel F., Cao J., Kim M., Sweredoski M.J., Hess S., Leitner A., Aebersold R., Peter M., Deshaies R.J., Enchev R.I. (2016). Structural and kinetic analysis of the COP9-Signalosome activation and the cullin-RING ubiquitin ligase deneddylation cycle. Elife.

[b0140] Nguyen T.H.D., Galej W.P., Bai X.C., Oubridge C., Newman A.J., Scheres S.H.W., Nagai K. (2016). Cryo-EM structure of the yeast U4/U6.U5 tri-snRNP at 3.7 A resolution. Nature.

[b0145] Nie Y., Bellon-Echeverria I., Trowitzsch S., Bieniossek C., Berger I. (2014). Multiprotein complex production in insect cells by using polyproteins. Methods Mol. Biol..

[b0150] Pijlman G.P., van Schijndel J.E., Vlak J.M. (2003). Spontaneous excision of BAC vector sequences from bacmid-derived baculovirus expression vectors upon passage in insect cells. J. Gen. Virol..

[b0155] Reck-Peterson S.L. (2015). Dynactin revealed. Nat. Struct. Mol. Biol..

[b0160] Sawicki S.G., Sawicki D.L., Siddell S.G. (2007). A contemporary view of coronavirus transcription. J. Virol..

[b0165] Scheres S.H. (2012). RELION: implementation of a Bayesian approach to cryo-EM structure determination. J. Struct. Biol..

[b0175] Tang G., Peng L., Baldwin P.R., Mann D.S., Jiang W., Rees I., Ludtke S.J. (2007). EMAN2: an extensible image processing suite for electron microscopy. J. Struct. Biol..

[b0180] Urnavicius L., Zhang K., Diamant A.G., Motz C., Schlager M.A., Yu M., Patel N.A., Robinson C.V., Carter A.P. (2015). The structure of the dynactin complex and its interaction with dynein. Science (New York, N.Y).

[b0185] van Oers M.M. (2011). Opportunities and challenges for the baculovirus expression system. J. Invertebr. Pathol..

[b0190] Vijayachandran L.S., Viola C., Garzoni F., Trowitzsch S., Bieniossek C., Chaillet M., Schaffitzel C., Busso D., Romier C., Poterszman A., Richmond T.J., Berger I. (2011). Robots, pipelines, polyproteins: enabling multiprotein expression in prokaryotic and eukaryotic cells. J. Struct. Biol..

[b0195] Wan L.C., Maisonneuve P., Szilard R.K., Lambert J.P., Ng T.F., Manczyk N., Huang H., Laister R., Caudy A.A., Gingras A.C., Durocher D., Sicheri F. (2017). Proteomic analysis of the human KEOPS complex identifies C14ORF142 as a core subunit homologous to yeast Gon7. Nucl. Acids Res..

[b0200] Wang S., Zhai Y., Pang X., Niu T., Ding Y.H., Dong M.Q., Hsu V.W., Sun Z., Sun F. (2016). Structural characterization of coatomer in its cytosolic state. Protein Cell.

[b0205] Wei R., Wang X., Zhang Y., Mukherjee S., Zhang L., Chen Q., Huang X., Jing S., Liu C., Li S., Wang G., Xu Y., Zhu S., Williams A.J., Sun F., Yin C.C. (2016). Structural insights into Ca(2+)-activated long-range allosteric channel gating of RyR1. Cell Res..

[b0210] Wei X., Su X., Cao P., Liu X., Chang W., Li M., Zhang X., Liu Z. (2016). Structure of spinach photosystem II-LHCII supercomplex at 3.2 A resolution. Nature.

[b0215] Weissmann F., Peters J.M. (2018). Expressing multi-subunit complexes using biGBac. Methods Mol. Biol..

[b0220] Weissmann F., Petzold G., VanderLinden R., Huis In 't Veld P.J., Brown N.G., Lampert F., Westermann S., Stark H., Schulman B.A., Peters J.M. (2016). biGBac enables rapid gene assembly for the expression of large multisubunit protein complexes. Proc. Natl. Acad. Sci. U.S.A..

[b0225] Wu B., Guo W. (2015). The exocyst at a glance. J. Cell Sci..

[b0230] Yan C., Hang J., Wan R., Huang M., Wong C.C., Shi Y. (2015). Structure of a yeast spliceosome at 3.6-angstrom resolution. Science (New York, N.Y).

[b0235] Yan Z., Bai X., Yan C., Wu J., Li Z., Xie T., Peng W., Yin C., Li X., Scheres S.H.W., Shi Y., Yan N. (2015). Structure of the rabbit ryanodine receptor RyR1 at near-atomic resolution. Nature.

[b0240] Yoo H.Y., Kumagai A., Shevchenko A., Shevchenko A., Dunphy W.G. (2009). The Mre11-Rad50-Nbs1 complex mediates activation of TopBP1 by ATM. Mol. Biol. Cell.

[b0245] Zhang K. (2016). Gctf: real-time CTF determination and correction. J. Struct. Biol..

[b0250] Zhang K., Foster H.E., Rondelet A., Lacey S.E., Bahi-Buisson N., Bird A.W., Carter A.P. (2017). Cryo-EM reveals how human cytoplasmic dynein is auto-inhibited and activated. Cell.

[b0255] Zheng N., Schulman B.A., Song L., Miller J.J., Jeffrey P.D., Wang P., Chu C., Koepp D.M., Elledge S.J., Pagano M., Conaway R.C., Conaway J.W., Harper J.W., Pavletich N.P. (2002). Structure of the Cul1-Rbx1-Skp1-F boxSkp2 SCF ubiquitin ligase complex. Nature.

[b0260] Ziebuhr J., Snijder E.J., Gorbalenya A.E. (2000). Virus-encoded proteinases and proteolytic processing in the Nidovirales. J. Gen. Virol..

