# Supplementary material for: SmartBac, a new baculovirus system for large protein complex production
Source: J Struct Biol X. 2019 Feb 10;1:100003. doi: 10.1016/j.yjsbx.2019.100003 (PMC7173262; doi:10.1016/j.yjsbx.2019.100003)
Supplement: Supplementary Data 1 [file mmc1.docx]

**Supplementary Information**

**Large protein complex production using the Smartbac System--- Strategies and Applications**

Yujia Zhai ^1, 2, *^, Danyang Zhang ^1, 2^, Leiye Yu ^1, 2^, Fang Sun ^1, 2^, Fei Sun ^1,2,3, *^

^1^ National Key Laboratory of Biomacromolecules, CAS Center for Excellence in Biomacromolecules, Institute of Biophysics, Chinese Academy of Sciences, Beijing 100101, China.

^2^ School of Life Sciences, University of Chinese Academy of Sciences, Beijing, China.

^3^ Center for Biological Imaging, Core Facilities for Protein Science, Institute of Biophysics, CAS, Beijing, China.

^*^ Correspondence should be addressed to Y.Z. ([yujia@ibp.ac.cn](mailto:yujia@ibp.ac.cn)) or F.S. ([feisun@ibp.ac.cn](mailto:feisun@ibp.ac.cn))

**Keywords**

Baculovirus system; Cre recombination; Gibbson assembly; Vectors; Protein complex production.

**Supplementary Materials and Methods**

**S1. Nucleotide sequences of the Smartbac vectors**

---> 4V1G: 7187bp

5’-GATGCCCTGCGTAAGCGGGTGTGGGCGGACAATAAAGTCTTAAACTGAACAAAATAGATCTAAACTATGACAATAAAGTCTTAAACTAGACAGAATAGTTGTAAACTGAAATCAGTCCAGTTATGCTGTGAAAAAGCATACTGGACTTTTGTTATGGCTAAAGCAAACTCTTCATTTTCTGAAGTGCAAATTGCCCGTCGTATTAAAGAGGGGCGTGGCCAAGGGCATGTAAAGACTATATTCGCGGCGTTGTGACAATTTACCGAACAACTCCGCGGCCGGGAAGCCGATCTCGGCTTGAACGAATTGTTAGGTGGCGGTACTTGGGTCGATATCAAAGTGCATCACTTCTTCCCGTATGCCCAACTTTGTATAGAGAGCCACTGCGGGATCGTCACCGTAATCTGCTTGCACGTAGATCACATAAGCACCAAGCGCGTTGGCCTCATGCTTGAGGAGATTGATGAGCGCGGTGGCAATGCCCTGCCTCCGGTGCTCGCCGGAGACTGCGAGATCATAGATATAGATCTCACTACGCGGCTGCTCAAACTTGGGCAGAACGTAAGCCGCGAGAGCGCCAACAACCGCTTCTTGGTCGAAGGCAGCAAGCGCGATGAATGTCTTACTACGGAGCAAGTTCCCGAGGTAATCGGAGTCCGGCTGATGTTGGGAGTAGGTGGCTACGTCTCCGAACTCACGACCGAAAAGATCAAGAGCAGCCCGCATGGATTTGACTTGGTCAGGGCCGAGCCTACATGTGCGAATGATGCCCATACTTGAGCCACCTAACTTTGTTTTAGGGCGACTGCCCTGCTGCGTAACATCGTTGCTGCTGCGTAACATCGTTGCTGCTCCATAACATCAAACATCGACCCACGGCGTAACGCGCTTGCTGCTTGGATGCCCGAGGCATAGACTGTACAAAAAAACAGTCATAACAAGCCATGAAAACCGCCACTGCGCCGTTACCACCGCTGCGTTCGGTCAAGGTTCTGGACCAGTTGCGTGAGCGCATACGCTACTTGCATTACAGTTTACGAACCGAACAGGCTTATGTCAACTGGGTTCGTGCCTTCATCCGTTTCCACGGTGTGCGTCACCCGGCAACCTTGGGCAGCAGCGAAGTCGCCATAACTTCGTATAGCATACATTATACGAAGTTATCTGTAACTATAACGGTCCTAAGGTAGCGAGTTTAAACACTAGTTCGCGACCTACTCCGGAATATTAATAGGTTGCTGATATCGGGAGTTCAGTCGTCGAATGCAAAGCGTAAAAAATATTAATAAGGTAAAAATTACAGCTACATAAATTACACAATTTAAACGGATCGATCATATGGCTTATCCTTACGACGTGCCTGACTACGCCGGAGAGAGCTTGTTTAAGGGGCCGCGTGATTACAACCCTATATCGAGCACCATTTGTCATTTGACGAATGAATCTGATGGGCACACAACATCGTTGTATGGTATTGGATTTGGTCCCTTCATCATTACAAACAAGCACTTGTTTAGAAGAAATAATGGAACACTGTTGGTCCAATCACTACATGGTGTATTCAAGGTCAAGAACACCACGACTTTGCAACAACACCTCATTGATGGGAGGGACATGATAATTATTCGCATGCCTAAGGATTTCCCACCATTTCCTCAAAAGCTGAAATTTAGAGAGCCACAAAGGGAAGAGAGAATATGTCTTGTGACAACCAACTTCCAAACTAAGAGCATGTCTAGCATGGTGTCAGACACTTCTTGCACATTCCCTTCATCTGATGGCATATTCTGGAAGCATTGGATTCAAACCAAGGATGGGCAGTGTGGCAGTCCATTAGTATCAACTAGAGATGGGTTCATTGTTGGTATACACTCAGCATCGAATTTCACCAACACAAACAATTATTTCACAAGCGTGCCGAAAAACTTCATGGAATTGTTGACAAATCAGGAGGCGCAGCAGTGGGTTAGTGGTTGGCGATTAAATGCTGACTCAGTATTGTGGGGGGGCCATAAAGTTTTCATGGTGAAACCTGAAGAGCCTTTTCAGCCAGTTAAGGAAGCGACTCAACTCATGAATGAATTGGTGTACTCGCAAGAAAACCTGTACTTCCAGTCAGCCTGGAGCCATCCGCAATTTGAAAAAGGTGGCGGGTCCGGCGGAGGTAGCGGCGGAGGTTCTTGGTCTCACCCTCAGTTCGAGAAGGATGACGATGATAAAACCATGGGATCCCTAGGTACCGCGGCCGCGCGCGTTGGCCGATTCATTAATGCAGCTGGCACGACAGGTTTCCCGACTGGAAAGCGGGCAGTGAGCGCAACGCAATTAATGTGAGTTAGCTCACTCATTAGGCACCCCAGGCTTTACACTTTATGCTTCCGGCTCGTATGTTGTGTGGAATTGTGAGCGGATAACAATTTCACACAGGAAACAGCTATGACCATGATTACGCCAAGCTATTTAGGTGACGCGTTAGAATACTCAAGCTATGCATCATCTTTGGTTCCGTCATCGGACCCATTAGTAACGGCCGCCAGTGTGCTGGAGTTTTGTAGATACCCATCACACTGGCGTCCACTGGAACATGCAAGTAGAGGGCCCAATTCGCCCTATAGTGAGTCGTATTACAATTCACTGGCCGTCGTTTTACAACGTCGTGACTGGGAAAACCCTGGCGTTACCCAACTTAATCGCCTTGCAGCACATCCCCCTTTCGCCAGCTGGCGTAATAGCGAAGAGGCCCGCACCGATCGCCCTTCCCAACAGTTGCGCAGCCTATACGTACGGTAACTGACTAAGAATTCCGATTACAAAGACGATGACGACAAGGGCTCGAGTGAAAATTTGTATTTTCAAAGCTCGTCGACGGTGAGCAAGGGCGAGGAGCTGTTCACCGGGGTGGTGCCCATCCTGGTCGAGCTGGACGGCGACGTAAACGGCCACAAGTTCAGCGTGTCCGGCGAGGGCGAGGGCGATGCCACCTACGGCAAGCTGACCCTGAAGTTCATCTGCACCACCGGCAAGCTGCCCGTGCCCTGGCCCACCCTCGTGACCACCCTGACCTACGGCGTGCAGTGCTTCAGCCGCTACCCCGACCACATGAAGCAGCACGACTTCTTCAAGTCCGCCATGCCCGAAGGCTACGTCCAGGAGCGCACCATCTTCTTCAAGGACGACGGCAACTACAAGACCCGCGCCGAGGTGAAGTTCGAGGGCGACACCCTGGTGAACCGCATCGAGCTGAAGGGCATCGACTTCAAGGAGGACGGCAACATCCTGGGGCACAAGCTGGAGTACAACTACAACAGCCACAACGTCTATATCATGGCCGACAAGCAGAAGAACGGCATCAAGGTGAACTTCAAGATCCGCCACAACATCGAGGACGGCAGCGTGCAGCTCGCCGACCACTACCAGCAGAACACCCCCATCGGCGACGGCCCCGTGCTGCTGCCCGACAACCACTACCTGAGCACCCAGTCCGCCCTGAGCAAAGACCCCAACGAGAAGCGCGATCACATGGTCCTGCTGGAGTTCGTGACCGCCGCCGGGATCACTCTCGGCATGGACGAGCTGTACAAGTAACTGACTAAAAGCTTCGAAAGGAAGCTGAGTTGGCTGCTGCCACCGCTGAGCAATAACTAGCATAACCCCTTGGGGCCTCTAAACGGGTCTTGAGGGGTTTTTTGCTGAAAGGAGGAACTATCCTCAGGGTCGAGAAGTACTAGAGGATCATAATCAGCCATACCACATTTGTAGAGGTTTTACTTGCTTTAAAAAACCTCCCACACCTCCCCCTGAACCTGAAACATAAAATGAATGCAATTGTTGTTGTTAACTTGTTTATTGCAGCTTATAATGGTTACAAATAAAGCAATAGCATCACAAATTTCACAAATAAAGCATTTTTTTCACTGCATTCTAGTTGTGGTTTGTCCAAACTCATCAATGTATCTTATCATGTCTGGATCTGATCACTGCTTGAGCCTAGGAGATCCGAACCAGATAAGTGAAATCTAGTTCCAAACTATTTTGTCATTTTTAATTTTCGTATTAGCTTACGACGCTACACCCAGTTCCCATCTATTTTGTCACTCTTCCCTAAATAATCCTTAAAAACTCCATTTCCACCCCTCCCAGTTCCCAACTATTTTGTCCGCCCACAGCGGGGCATTTTTCTTCCTGTTATGTTTTTAATCAAACATCCTGCCAACTCCATGTGACAAACCGTCATCTTCGGCTACTTTTTCTCTGTCACAGAATGAAAATTTTTCTGTCATCTCTTCGTTATTAATGTTTGTAATTGACTGAATATCAACGCTTATTTGCAGCCTGAATGGCGAATGGGACGCGCCCTGTAGCGGCGCATTAAGCGCGGCGGGTGTGGTGGTTACGCGCAGCGTGACCGCTACACTTGCCAGCGCCCTAGCGCCCGCTCCTTTCGCTTTCTTCCCTTCCTTTCTCGCCACGTTCGCCGGCTTTCCCCGTCAAGCTCTAAATCGGGGGCTCCCTTTAGGGTTCCGATTTAGTGCTTTACGGCACCTCGACCCCAAAAAACTTGATTAGGGTGATGGTTCACGTAGTGGGCCATCGCCCTGATAGACGGTTTTTCGCCCTTTGACGTTGGAGTCCACGTTCTTTAATAGTGGACTCTTGTTCCAAACTGGAACAACACTCAACCCTATCTCGGTCTATTCTTTTGATTTATAAGGGATTTTGCCGATTTCGGCCTATTGGTTAAAAAATGAGCTGATTTAACAAAAATTTAACGCGAATTTTAACAAAATATTAACGTTTACAATTTCAGGTGGCACTTTTCGGGGAAATGTGCGCGGAACCCCTATTTGTTTATTTTTCTAAATACATTCAAATATGTATCCGCTCATGAGACAATAACCCTGATAAATGCTTCAATAATATTGAAAAAGGAAGAGTATGAGTATTCAACATTTCCGTGTCGCCCTTATTCCCTTTTTTGCGGCATTTTGCCTTCCTGTTTTTGCTCACCCAGAAACGCTGGTGAAAGTAAAAGATGCTGAAGATCAGTTGGGTGCACGAGTGGGTTACATCGAACTGGATCTCAACAGCGGTAAGATCCTTGAGAGTTTTCGCCCCGAAGAACGTTTTCCAATGATGAGCACTTTTAAAGTTCTGCTATGTGGCGCGGTATTATCCCGTATTGACGCCGGGCAAGAGCAACTCGGTCGCCGCATACACTATTCTCAGAATGACTTGGTTGAGTACTCACCAGTCACAGAAAAGCATCTTACGGATGGCATGACAGTAAGAGAATTATGCAGTGCTGCCATAACCATGAGTGATAACACTGCGGCCAACTTACTTCTGACAACGATCGGAGGACCGAAGGAGCTAACCGCTTTTTTGCACAACATGGGGGATCATGTAACTCGCCTTGATCGTTGGGAACCGGAGCTGAATGAAGCCATACCAAACGACGAGCGTGACACCACGATGCCTGTAGCAATGGCAACAACGTTGCGCAAACTATTAACTGGCGAACTACTTACTCTAGCTTCCCGGCAACAATTAATAGACTGGATGGAGGCGGATAAAGTTGCAGGACCACTTCTGCGCTCGGCCCTTCCGGCTGGCTGGTTTATTGCTGATAAATCTGGAGCCGGTGAGCGTGGGTCTCGCGGTATCATTGCAGCACTGGGGCCAGATGGTAAGCCCTCCCGTATCGTAGTTATCTACACGACGGGGAGTCAGGCAACTATGGATGAACGAAATAGACAGATCGCTGAGATAGGTGCCTCACTGATTAAGCATTGGTAACTGTCAGACCAAGTTTACTCATATATACTTTAGATTGATTTAAAACTTCATTTTTAATTTAAAAGGATCTAGGTGAAGATCCTTTTTGATAATCTCATGACCAAAATCCCTTAACGTGAGTTTTCGTTCCACTGAGCGTCAGACCGCGGGGCATGACTAACATGAGAATTACAACTTATATCGTATGGGGCTGACTTCAGGTGCTACATTTGAAGAGATAAATTGCACTGAAATCTAGAAATATTTTATCTGATTAATAAGATGATCTTCTTGAGATCGTTTTGGTCTGCGCGTAATCTCTTGCTCTGAAAACGGAAAAAACCGCCTTGCAGGGCGGTTTTTCGAAGGTTCTCTGAGCTACCAACTCTTTGAACCGAGGTAACTGGCTTGGAGGAGCGCAGTCACCAAAACTTGTCCTTTCAGTTTAGCCTTAACCGGCGCATGACTTCAAGACTAACTCCTCTAAATCAATTACCAGTGGCTGCTGCCAGTGGTGCTTTTGCATGTCTTTCCGGGTTGGACTCAAGACGATAGTTACCGGATAAGGCGCAGCGGTCGGACTGAACGGGGGGTTCGTGCATACAGTCCAGCTTGGAGCGAACTGCCTACCCGGAACTGAGTGTCAGGCGTGGAATGAGACAAACGCGGCCATAACAGCGGAATGACACCGGTAAACCGAAAGGCAGGAACAGGAGAGCGCACGAGGGAGCCGCCAGGGGAAACGCCTGGTATCTTTATAGTCCTGTCGGGTTTCGCCACCACTGATTTGAGCGTCAGATTTCGTGATGCTTGTCAGGGGGGCGGAGCCTATGGAAAAACGGCTTTGCCGCGGCCCTCTCACTTCCCTGTTAAGTATCTTCCTGGCATCTTCCAGGAAATCTCCGCCCCGTTCGTAAGCCATTTCCGCTCGCCGCAGTCGAACGACCGAGCGTAGCGAGTCAGTGAGCGAGGAAGCGGAATATATCCTGTATCACATATTCTGCTGACGCACCGGTGCAGCCTTTTTTCTCCTGCCACATGAAGCACTTCACTGACACCCTCATCAGTGCCAACATAGTAAGCCAGTATACACTCCGCTAGCGCTGATGTCCGGCGGTGCTTTTGCCGTTACGCACCACCCCGTCAGTAGCTGAACAGGAGGGACAGCTGATAGAAACAGAAGCCAGTTCTTTCCTGCGTTATCCCCTGATTCTGTGGATAACCGTATTACCGCCTTTGAGTGAGCTGATACCGCTCGCCGCAGCCGAACGACCGAGCGCAGCGAGTCAGTGAGCGAGGAAGCGGAAGAGCGCCTGATGCGGTATTTTCTCCTTACGCATCTGTGCGGTATTTCACACCGCATAGACCAGCCGCGTAACCTGGCAAAATCGGTTACGGTTGAGTAATAAATG-3’

---> 4V1R:7187bp

5’-GATGCCCTGCGTAAGCGGGTGTGGGCGGACAATAAAGTCTTAAACTGAACAAAATAGATCTAAACTATGACAATAAAGTCTTAAACTAGACAGAATAGTTGTAAACTGAAATCAGTCCAGTTATGCTGTGAAAAAGCATACTGGACTTTTGTTATGGCTAAAGCAAACTCTTCATTTTCTGAAGTGCAAATTGCCCGTCGTATTAAAGAGGGGCGTGGCCAAGGGCATGTAAAGACTATATTCGCGGCGTTGTGACAATTTACCGAACAACTCCGCGGCCGGGAAGCCGATCTCGGCTTGAACGAATTGTTAGGTGGCGGTACTTGGGTCGATATCAAAGTGCATCACTTCTTCCCGTATGCCCAACTTTGTATAGAGAGCCACTGCGGGATCGTCACCGTAATCTGCTTGCACGTAGATCACATAAGCACCAAGCGCGTTGGCCTCATGCTTGAGGAGATTGATGAGCGCGGTGGCAATGCCCTGCCTCCGGTGCTCGCCGGAGACTGCGAGATCATAGATATAGATCTCACTACGCGGCTGCTCAAACTTGGGCAGAACGTAAGCCGCGAGAGCGCCAACAACCGCTTCTTGGTCGAAGGCAGCAAGCGCGATGAATGTCTTACTACGGAGCAAGTTCCCGAGGTAATCGGAGTCCGGCTGATGTTGGGAGTAGGTGGCTACGTCTCCGAACTCACGACCGAAAAGATCAAGAGCAGCCCGCATGGATTTGACTTGGTCAGGGCCGAGCCTACATGTGCGAATGATGCCCATACTTGAGCCACCTAACTTTGTTTTAGGGCGACTGCCCTGCTGCGTAACATCGTTGCTGCTGCGTAACATCGTTGCTGCTCCATAACATCAAACATCGACCCACGGCGTAACGCGCTTGCTGCTTGGATGCCCGAGGCATAGACTGTACAAAAAAACAGTCATAACAAGCCATGAAAACCGCCACTGCGCCGTTACCACCGCTGCGTTCGGTCAAGGTTCTGGACCAGTTGCGTGAGCGCATACGCTACTTGCATTACAGTTTACGAACCGAACAGGCTTATGTCAACTGGGTTCGTGCCTTCATCCGTTTCCACGGTGTGCGTCACCCGGCAACCTTGGGCAGCAGCGAAGTCGCCATAACTTCGTATAGCATACATTATACGAAGTTATCTGTAACTATAACGGTCCTAAGGTAGCGAGTTTAAACACTAGTTCGCGACCTACTCCGGAATATTAATAGGTTGCTGATATCGGGAGTTCAGTCGTCGAATGCAAAGCGTAAAAAATATTAATAAGGTAAAAATTACAGCTACATAAATTACACAATTTAAACGGATCGATCATATGGCTTATCCTTACGACGTGCCTGACTACGCCGGAGAGAGCTTGTTTAAGGGGCCGCGTGATTACAACCCTATATCGAGCACCATTTGTCATTTGACGAATGAATCTGATGGGCACACAACATCGTTGTATGGTATTGGATTTGGTCCCTTCATCATTACAAACAAGCACTTGTTTAGAAGAAATAATGGAACACTGTTGGTCCAATCACTACATGGTGTATTCAAGGTCAAGAACACCACGACTTTGCAACAACACCTCATTGATGGGAGGGACATGATAATTATTCGCATGCCTAAGGATTTCCCACCATTTCCTCAAAAGCTGAAATTTAGAGAGCCACAAAGGGAAGAGAGAATATGTCTTGTGACAACCAACTTCCAAACTAAGAGCATGTCTAGCATGGTGTCAGACACTTCTTGCACATTCCCTTCATCTGATGGCATATTCTGGAAGCATTGGATTCAAACCAAGGATGGGCAGTGTGGCAGTCCATTAGTATCAACTAGAGATGGGTTCATTGTTGGTATACACTCAGCATCGAATTTCACCAACACAAACAATTATTTCACAAGCGTGCCGAAAAACTTCATGGAATTGTTGACAAATCAGGAGGCGCAGCAGTGGGTTAGTGGTTGGCGATTAAATGCTGACTCAGTATTGTGGGGGGGCCATAAAGTTTTCATGGTGAAACCTGAAGAGCCTTTTCAGCCAGTTAAGGAAGCGACTCAACTCATGAATGAATTGGTGTACTCGCAAGAAAACCTGTACTTCCAGTCAGCCTGGAGCCATCCGCAATTTGAAAAAGGTGGCGGGTCCGGCGGAGGTAGCGGCGGAGGTTCTTGGTCTCACCCTCAGTTCGAGAAGGATGACGATGATAAAACCATGGGATCCCTAGGTACCGCGGCCGCGCGCGTTGGCCGATTCATTAATGCAGCTGGCACGACAGGTTTCCCGACTGGAAAGCGGGCAGTGAGCGCAACGCAATTAATGTGAGTTAGCTCACTCATTAGGCACCCCAGGCTTTACACTTTATGCTTCCGGCTCGTATGTTGTGTGGAATTGTGAGCGGATAACAATTTCACACAGGAAACAGCTATGACCATGATTACGCCAAGCTATTTAGGTGACGCGTTAGAATACTCAAGCTATGCATCATCTTTGGTTCCGTCATCGGACCCATTAGTAACGGCCGCCAGTGTGCTGGAGTTTTGTAGATACCCATCACACTGGCGTCCACTGGAACATGCAAGTAGAGGGCCCAATTCGCCCTATAGTGAGTCGTATTACAATTCACTGGCCGTCGTTTTACAACGTCGTGACTGGGAAAACCCTGGCGTTACCCAACTTAATCGCCTTGCAGCACATCCCCCTTTCGCCAGCTGGCGTAATAGCGAAGAGGCCCGCACCGATCGCCCTTCCCAACAGTTGCGCAGCCTATACGTACGGTAACTGACTAAGAATTCCGAGCAAAAGTTGATTAGCGAAGAAGACTTAGGCTCGAGTGAAAATTTGTATTTTCAAAGCTCGTCGACGGTGTCTAAGGGCGAAGAGCTGATTAAGGAGAACATGCACATGAAGCTGTACATGGAGGGCACCGTGAACAACCACCACTTCAAGTGCACATCCGAGGGCGAAGGCAAGCCCTACGAGGGCACCCAGACCATGAGAATCAAGGTGGTCGAGGGCGGCCCTCTCCCCTTCGCCTTCGACATCCTGGCTACCAGCTTCATGTACGGCAGCAGAACCTTCATCAACCACACCCAGGGCATCCCCGACTTCTTTAAGCAGTCCTTCCCTGAGGGCTTCACATGGGAGAGAGTCACCACATACGAAGACGGGGGCGTGCTGACCGCTACCCAGGACACCAGCCTCCAGGACGGCTGCCTCATCTACAACGTCAAGATCAGAGGGGTGAACTTCCCATCCAACGGCCCTGTGATGCAGAAGAAAACACTCGGCTGGGAGGCCAACACCGAGATGCTGTACCCCGCTGACGGCGGCCTGGAAGGCAGAAGCGACATGGCCCTGAAGCTCGTGGGCGGGGGCCACCTGATCTGCAACTTCAAGACCACATACAGATCCAAGAAACCCGCTAAGAACCTCAAGATGCCCGGCGTCTACTATGTGGACCACAGACTGGAAAGAATCAAGGAGGCCGACAAAGAGACCTACGTCGAGCAGCACGAGGTGGCTGTGGCCAGATACTGCGACCTCCCTAGCAAACTGGGGCACAAACTTAATTAACTGACTAAAAGCTTCGAAAGGAAGCTGAGTTGGCTGCTGCCACCGCTGAGCAATAACTAGCATAACCCCTTGGGGCCTCTAAACGGGTCTTGAGGGGTTTTTTGCTGAAAGGAGGAACTATCCTCAGGGTCGAGAAGTACTAGAGGATCATAATCAGCCATACCACATTTGTAGAGGTTTTACTTGCTTTAAAAAACCTCCCACACCTCCCCCTGAACCTGAAACATAAAATGAATGCAATTGTTGTTGTTAACTTGTTTATTGCAGCTTATAATGGTTACAAATAAAGCAATAGCATCACAAATTTCACAAATAAAGCATTTTTTTCACTGCATTCTAGTTGTGGTTTGTCCAAACTCATCAATGTATCTTATCATGTCTGGATCTGATCACTGCTTGAGCCTAGGAGATCCGAACCAGATAAGTGAAATCTAGTTCCAAACTATTTTGTCATTTTTAATTTTCGTATTAGCTTACGACGCTACACCCAGTTCCCATCTATTTTGTCACTCTTCCCTAAATAATCCTTAAAAACTCCATTTCCACCCCTCCCAGTTCCCAACTATTTTGTCCGCCCACAGCGGGGCATTTTTCTTCCTGTTATGTTTTTAATCAAACATCCTGCCAACTCCATGTGACAAACCGTCATCTTCGGCTACTTTTTCTCTGTCACAGAATGAAAATTTTTCTGTCATCTCTTCGTTATTAATGTTTGTAATTGACTGAATATCAACGCTTATTTGCAGCCTGAATGGCGAATGGGACGCGCCCTGTAGCGGCGCATTAAGCGCGGCGGGTGTGGTGGTTACGCGCAGCGTGACCGCTACACTTGCCAGCGCCCTAGCGCCCGCTCCTTTCGCTTTCTTCCCTTCCTTTCTCGCCACGTTCGCCGGCTTTCCCCGTCAAGCTCTAAATCGGGGGCTCCCTTTAGGGTTCCGATTTAGTGCTTTACGGCACCTCGACCCCAAAAAACTTGATTAGGGTGATGGTTCACGTAGTGGGCCATCGCCCTGATAGACGGTTTTTCGCCCTTTGACGTTGGAGTCCACGTTCTTTAATAGTGGACTCTTGTTCCAAACTGGAACAACACTCAACCCTATCTCGGTCTATTCTTTTGATTTATAAGGGATTTTGCCGATTTCGGCCTATTGGTTAAAAAATGAGCTGATTTAACAAAAATTTAACGCGAATTTTAACAAAATATTAACGTTTACAATTTCAGGTGGCACTTTTCGGGGAAATGTGCGCGGAACCCCTATTTGTTTATTTTTCTAAATACATTCAAATATGTATCCGCTCATGAGACAATAACCCTGATAAATGCTTCAATAATATTGAAAAAGGAAGAGTATGAGTATTCAACATTTCCGTGTCGCCCTTATTCCCTTTTTTGCGGCATTTTGCCTTCCTGTTTTTGCTCACCCAGAAACGCTGGTGAAAGTAAAAGATGCTGAAGATCAGTTGGGTGCACGAGTGGGTTACATCGAACTGGATCTCAACAGCGGTAAGATCCTTGAGAGTTTTCGCCCCGAAGAACGTTTTCCAATGATGAGCACTTTTAAAGTTCTGCTATGTGGCGCGGTATTATCCCGTATTGACGCCGGGCAAGAGCAACTCGGTCGCCGCATACACTATTCTCAGAATGACTTGGTTGAGTACTCACCAGTCACAGAAAAGCATCTTACGGATGGCATGACAGTAAGAGAATTATGCAGTGCTGCCATAACCATGAGTGATAACACTGCGGCCAACTTACTTCTGACAACGATCGGAGGACCGAAGGAGCTAACCGCTTTTTTGCACAACATGGGGGATCATGTAACTCGCCTTGATCGTTGGGAACCGGAGCTGAATGAAGCCATACCAAACGACGAGCGTGACACCACGATGCCTGTAGCAATGGCAACAACGTTGCGCAAACTATTAACTGGCGAACTACTTACTCTAGCTTCCCGGCAACAATTAATAGACTGGATGGAGGCGGATAAAGTTGCAGGACCACTTCTGCGCTCGGCCCTTCCGGCTGGCTGGTTTATTGCTGATAAATCTGGAGCCGGTGAGCGTGGGTCTCGCGGTATCATTGCAGCACTGGGGCCAGATGGTAAGCCCTCCCGTATCGTAGTTATCTACACGACGGGGAGTCAGGCAACTATGGATGAACGAAATAGACAGATCGCTGAGATAGGTGCCTCACTGATTAAGCATTGGTAACTGTCAGACCAAGTTTACTCATATATACTTTAGATTGATTTAAAACTTCATTTTTAATTTAAAAGGATCTAGGTGAAGATCCTTTTTGATAATCTCATGACCAAAATCCCTTAACGTGAGTTTTCGTTCCACTGAGCGTCAGACCGCGGGGCATGACTAACATGAGAATTACAACTTATATCGTATGGGGCTGACTTCAGGTGCTACATTTGAAGAGATAAATTGCACTGAAATCTAGAAATATTTTATCTGATTAATAAGATGATCTTCTTGAGATCGTTTTGGTCTGCGCGTAATCTCTTGCTCTGAAAACGGAAAAAACCGCCTTGCAGGGCGGTTTTTCGAAGGTTCTCTGAGCTACCAACTCTTTGAACCGAGGTAACTGGCTTGGAGGAGCGCAGTCACCAAAACTTGTCCTTTCAGTTTAGCCTTAACCGGCGCATGACTTCAAGACTAACTCCTCTAAATCAATTACCAGTGGCTGCTGCCAGTGGTGCTTTTGCATGTCTTTCCGGGTTGGACTCAAGACGATAGTTACCGGATAAGGCGCAGCGGTCGGACTGAACGGGGGGTTCGTGCATACAGTCCAGCTTGGAGCGAACTGCCTACCCGGAACTGAGTGTCAGGCGTGGAATGAGACAAACGCGGCCATAACAGCGGAATGACACCGGTAAACCGAAAGGCAGGAACAGGAGAGCGCACGAGGGAGCCGCCAGGGGAAACGCCTGGTATCTTTATAGTCCTGTCGGGTTTCGCCACCACTGATTTGAGCGTCAGATTTCGTGATGCTTGTCAGGGGGGCGGAGCCTATGGAAAAACGGCTTTGCCGCGGCCCTCTCACTTCCCTGTTAAGTATCTTCCTGGCATCTTCCAGGAAATCTCCGCCCCGTTCGTAAGCCATTTCCGCTCGCCGCAGTCGAACGACCGAGCGTAGCGAGTCAGTGAGCGAGGAAGCGGAATATATCCTGTATCACATATTCTGCTGACGCACCGGTGCAGCCTTTTTTCTCCTGCCACATGAAGCACTTCACTGACACCCTCATCAGTGCCAACATAGTAAGCCAGTATACACTCCGCTAGCGCTGATGTCCGGCGGTGCTTTTGCCGTTACGCACCACCCCGTCAGTAGCTGAACAGGAGGGACAGCTGATAGAAACAGAAGCCAGTTCTTTCCTGCGTTATCCCCTGATTCTGTGGATAACCGTATTACCGCCTTTGAGTGAGCTGATACCGCTCGCCGCAGCCGAACGACCGAGCGCAGCGAGTCAGTGAGCGAGGAAGCGGAAGAGCGCCTGATGCGGTATTTTCTCCTTACGCATCTGTGCGGTATTTCACACCGCATAGACCAGCCGCGTAACCTGGCAAAATCGGTTACGGTTGAGTAATAAATG-3’

---> 5V1TG: 7737bp

5’-GATGCCCTGCGTAAGCGGGTGTGGGCGGACAATAAAGTCTTAAACTGAACAAAATAGATCTAAACTATGACAATAAAGTCTTAAACTAGACAGAATAGTTGTAAACTGAAATCAGTCCAGTTATGCTGTGAAAAAGCATACTGGACTTTTGTTATGGCTAAAGCAAACTCTTCATTTTCTGAAGTGCAAATTGCCCGTCGTATTAAAGAGGGGCGTGGCCAAGGGCATGTAAAGACTATATTCGCGGCGTTGTGACAATTTACCGAACAACTCCGCGGCCGGGAAGCCGATCTCGGCTTGAACGAATTGTTAGGTGGCGGTACTTGGGTCGATATCAAAGTGCATCACTTCTTCCCGTATGCCCAACTTTGTATAGAGAGCCACTGCGGGATCGTCACCGTAATCTGCTTGCACGTAGATCACATAAGCACCAAGCGCGTTGGCCTCATGCTTGAGGAGATTGATGAGCGCGGTGGCAATGCCCTGCCTCCGGTGCTCGCCGGAGACTGCGAGATCATAGATATAGATCTCACTACGCGGCTGCTCAAACTTGGGCAGAACGTAAGCCGCGAGAGCGCCAACAACCGCTTCTTGGTCGAAGGCAGCAAGCGCGATGAATGTCTTACTACGGAGCAAGTTCCCGAGGTAATCGGAGTCCGGCTGATGTTGGGAGTAGGTGGCTACGTCTCCGAACTCACGACCGAAAAGATCAAGAGCAGCCCGCATGGATTTGACTTGGTCAGGGCCGAGCCTACATGTGCGAATGATGCCCATACTTGAGCCACCTAACTTTGTTTTAGGGCGACTGCCCTGCTGCGTAACATCGTTGCTGCTGCGTAACATCGTTGCTGCTCCATAACATCAAACATCGACCCACGGCGTAACGCGCTTGCTGCTTGGATGCCCGAGGCATAGACTGTACAAAAAAACAGTCATAACAAGCCATGAAAACCGCCACTGCGCCGTTACCACCGCTGCGTTCGGTCAAGGTTCTGGACCAGTTGCGTGAGCGCATACGCTACTTGCATTACAGTTTACGAACCGAACAGGCTTATGTCAACTGGGTTCGTGCCTTCATCCGTTTCCACGGTGTGCGTCACCCGGCAACCTTGGGCAGCAGCGAAGTCGCCATAACTTCGTATAGCATACATTATACGAAGTTATCTGTAACTATAACGGTCCTAAGGTAGCGAGTTTAAACGACGTCCTAGATTGGTTACTGGGCGATGAAGGTTTAGTCGGCAAATCGTCCAACGTTAGTGACAGCGTCAGCGGCAAGTTAATGCCTATCATTTTGTTGATAGGCGCGGTCTTGTTTTTAGGTTTAATATTTTATTTTATCTACAGATACATGATGAAAGGAGGGAAGGGAGGTGGTGTTGGCGCAGCAACGTCGCCAACTCCCATTGTTATTTCTATGCAAAACCCCACACCAACAACGGCCCCTCGATAATAAAAGACAAAAATAATATAAAATATATGTATAATTAATTAAATTCAAAATATATGTATAAGGCCGGCCTTAGTCAGTTACTTGTACAGCTCGTCCATGCCGAGAGTGATCCCGGCGGCGGTCACGAACTCCAGCAGGACCATGTGATCGCGCTTCTCGTTGGGGTCTTTGCTCAGGGCGGACTGGGTGCTCAGGTAGTGGTTGTCGGGCAGCAGCACGGGGCCGTCGCCGATGGGGGTGTTCTGCTGGTAGTGGTCGGCGAGCTGCACGCTGCCGTCCTCGATGTTGTGGCGGATCTTGAAGTTCACCTTGATGCCGTTCTTCTGCTTGTCGGCCATGATATAGACGTTGTGGCTGTTGTAGTTGTACTCCAGCTTGTGCCCCAGGATGTTGCCGTCCTCCTTGAAGTCGATGCCCTTCAGCTCGATGCGGTTCACCAGGGTGTCGCCCTCGAACTTCACCTCGGCGCGGGTCTTGTAGTTGCCGTCGTCCTTGAAGAAGATGGTGCGCTCCTGGACGTAGCCTTCGGGCATGGCGGACTTGAAGAAGTCGTGCTGCTTCATGTGGTCGGGGTAGCGGCTGAAGCACTGCACGCCGTAGGTCAGGGTGGTCACGAGGGTGGGCCAGGGCACGGGCAGCTTGCCGGTGGTGCAGATGAACTTCAGGGTCAGCTTGCCGTAGGTGGCATCGCCCTCGCCCTCGCCGGACACGCTGAACTTGTGGCCGTTTACGTCGCCGTCCAGCTCGACCAGGATGGGCACCACCCCGGTGAACAGCTCCTCGCCCTTGCTCATCCCGGGTGACTGGAAGTACAGGTTTTCTTGCGAGTACACCAATTCATTCATGAGTTGAGTCGCTTCCTTAACTGGCTGAAAAGGCTCTTCAGGTTTCACCATGAAAACTTTATGGCCCCCCCACAATACTGAGTCAGCATTTAATCGCCAACCACTAACCCACTGCTGCGCCTCCTGATTTGTCAACAATTCCATGAAGTTTTTCGGCACGCTTGTGAAATAATTGTTTGTGTTGGTGAAATTCGATGCTGAGTGTATACCAACAATGAACCCATCTCTAGTTGATACTAATGGACTGCCACACTGCCCATCCTTGGTTTGAATCCAATGCTTCCAGAATATGCCATCAGATGAAGGGAATGTGCAAGAAGTGTCTGACACCATGCTAGACATGCTCTTAGTTTGGAAGTTGGTTGTCACAAGACATATTCTCTCTTCCCTTTGTGGCTCTCTAAATTTCAGCTTTTGAGGAAATGGTGGGAAATCCTTAGGCATGCGAATAATTATCATGTCCCTCCCATCAATGAGGTGTTGTTGCAAAGTCGTGGTGTTCTTGACCTTGAATACACCATGTAGTGATTGGACCAACAGTGTTCCATTATTTCTTCTAAACAAGTGCTTGTTTGTAATGATGAAGGGACCAAATCCAATACCATACAACGATGTTGTGTGCCCATCAGATTCATTCGTCAAATGACAAATGGTGCTCGATATAGGGTTGTAATCACGCGGCCCCTTAAACAAGCTCTCTCCGGCGTAGTCAGGCACGTCGTAAGGATAAGCCATATTTAAATATATGCTTGCTTGTGTGTTCCTTATTGAAGCCTTGGTGTGACTGATTTACTAGTAGCGTTGAGGCGTCTTATATACCCGACCGTTATCTGGCCTACGTGACACAAGGCACGTTGTTAGATTAATAATCTTATCTTTTTATCTTAATTGATAAGATTATTTTTATCTGGCTGTTATAAAAACGGGATCATGAACACGGACGCTCAGTCGACAGATCTGTCGACGGTTTAAACACTAGTTCGCGACCTACTCCGGAATATTAATAGGTTGCTGATATCGGGAGTTCAGTCGTCGAATGCAAAGCGTAAAAAATATTAATAAGGTAAAAATTACAGCTACATAAATTACACAATTTAAACGGATCGATGAGCTCCATATGGCCTGGAGCCATCCGCAATTTGAAAAAGGTGGCGGGTCCGGCGGAGGTAGCGGCGGAGGTTCTTGGTCTCACCCTCAGTTCGAGAAGGATGACGATGATAAAACCATGGGATCCCTAGGTACCGCGGCCGCGCGCGTTGGCCGATTCATTAATGCAGCTGGCACGACAGGTTTCCCGACTGGAAAGCGGGCAGTGAGCGCAACGCAATTAATGTGAGTTAGCTCACTCATTAGGCACCCCAGGCTTTACACTTTATGCTTCCGGCTCGTATGTTGTGTGGAATTGTGAGCGGATAACAATTTCACACAGGAAACAGCTATGACCATGATTACGCCAAGCTATTTAGGTGACGCGTTAGAATACTCAAGCTATGCATCATCTTTGGTTCCGTCATCGGACCCATTAGTAACGGCCGCCAGTGTGCTGGAGTTTTGTAGATACCCATCACACTGGCGTCCACTGGAACATGCAAGTAGAGGGCCCAATTCGCCCTATAGTGAGTCGTATTACAATTCACTGGCCGTCGTTTTACAACGTCGTGACTGGGAAAACCCTGGCGTTACCCAACTTAATCGCCTTGCAGCACATCCCCCTTTCGCCAGCTGGCGTAATAGCGAAGAGGCCCGCACCGATCGCCCTTCCCAACAGTTGCGCAGCCTATACGTACGGTAACTGACTAAGAATTCCTTAAGCGGAGGCCTGCAGGGCTCGAGTTAACTGACTAAAAGCTTCGAAAGGAAGCTGAGTTGGCTGCTGCCACCGCTGAGCAATAACTAGCATAACCCCTTGGGGCCTCTAAACGGGTCTTGAGGGGTTTTTTGCTGAAAGGAGGAACTATCCTCAGGGTCGAGAAGTACTAGAGGATCATAATCAGCCATACCACATTTGTAGAGGTTTTACTTGCTTTAAAAAACCTCCCACACCTCCCCCTGAACCTGAAACATAAAATGAATGCAATTGTTGTTGTTAACTTGTTTATTGCAGCTTATAATGGTTACAAATAAAGCAATAGCATCACAAATTTCACAAATAAAGCATTTTTTTCACTGCATTCTAGTTGTGGTTTGTCCAAACTCATCAATGTATCTTATCATGTCTGGATCTGATCACTGCTTGAGCCTAGGAGATCCGAACCAGATAAGTGAAATCTAGTTCCAAACTATTTTGTCATTTTTAATTTTCGTATTAGCTTACGACGCTACACCCAGTTCCCATCTATTTTGTCACTCTTCCCTAAATAATCCTTAAAAACTCCATTTCCACCCCTCCCAGTTCCCAACTATTTTGTCCGCCCACAGCGGGGCATTTTTCTTCCTGTTATGTTTTTAATCAAACATCCTGCCAACTCCATGTGACAAACCGTCATCTTCGGCTACTTTTTCTCTGTCACAGAATGAAAATTTTTCTGTCATCTCTTCGTTATTAATGTTTGTAATTGACTGAATATCAACGCTTATTTGCAGCCTGAATGGCGAATGGGACGCGCCCTGTAGCGGCGCATTAAGCGCGGCGGGTGTGGTGGTTACGCGCAGCGTGACCGCTACACTTGCCAGCGCCCTAGCGCCCGCTCCTTTCGCTTTCTTCCCTTCCTTTCTCGCCACGTTCGCCGGCTTTCCCCGTCAAGCTCTAAATCGGGGGCTCCCTTTAGGGTTCCGATTTAGTGCTTTACGGCACCTCGACCCCAAAAAACTTGATTAGGGTGATGGTTCACGTAGTGGGCCATCGCCCTGATAGACGGTTTTTCGCCCTTTGACGTTGGAGTCCACGTTCTTTAATAGTGGACTCTTGTTCCAAACTGGAACAACACTCAACCCTATCTCGGTCTATTCTTTTGATTTATAAGGGATTTTGCCGATTTCGGCCTATTGGTTAAAAAATGAGCTGATTTAACAAAAATTTAACGCGAATTTTAACAAAATATTAACGTTTACAATTTCAGGTGGCACTTTTCGGGGAAATGTGCGCGGAACCCCTATTTGTTTATTTTTCTAAATACATTCAAATATGTATCCGCTCATGAGACAATAACCCTGATAAATGCTTCAATAATATTGAAAAAGGAAGAGTATGAGTATTCAACATTTCCGTGTCGCCCTTATTCCCTTTTTTGCGGCATTTTGCCTTCCTGTTTTTGCTCACCCAGAAACGCTGGTGAAAGTAAAAGATGCTGAAGATCAGTTGGGTGCACGAGTGGGTTACATCGAACTGGATCTCAACAGCGGTAAGATCCTTGAGAGTTTTCGCCCCGAAGAACGTTTTCCAATGATGAGCACTTTTAAAGTTCTGCTATGTGGCGCGGTATTATCCCGTATTGACGCCGGGCAAGAGCAACTCGGTCGCCGCATACACTATTCTCAGAATGACTTGGTTGAGTACTCACCAGTCACAGAAAAGCATCTTACGGATGGCATGACAGTAAGAGAATTATGCAGTGCTGCCATAACCATGAGTGATAACACTGCGGCCAACTTACTTCTGACAACGATCGGAGGACCGAAGGAGCTAACCGCTTTTTTGCACAACATGGGGGATCATGTAACTCGCCTTGATCGTTGGGAACCGGAGCTGAATGAAGCCATACCAAACGACGAGCGTGACACCACGATGCCTGTAGCAATGGCAACAACGTTGCGCAAACTATTAACTGGCGAACTACTTACTCTAGCTTCCCGGCAACAATTAATAGACTGGATGGAGGCGGATAAAGTTGCAGGACCACTTCTGCGCTCGGCCCTTCCGGCTGGCTGGTTTATTGCTGATAAATCTGGAGCCGGTGAGCGTGGGTCTCGCGGTATCATTGCAGCACTGGGGCCAGATGGTAAGCCCTCCCGTATCGTAGTTATCTACACGACGGGGAGTCAGGCAACTATGGATGAACGAAATAGACAGATCGCTGAGATAGGTGCCTCACTGATTAAGCATTGGTAACTGTCAGACCAAGTTTACTCATATATACTTTAGATTGATTTAAAACTTCATTTTTAATTTAAAAGGATCTAGGTGAAGATCCTTTTTGATAATCTCATGACCAAAATCCCTTAACGTGAGTTTTCGTTCCACTGAGCGTCAGACCGCGGGGCATGACTAACATGAGAATTACAACTTATATCGTATGGGGCTGACTTCAGGTGCTACATTTGAAGAGATAAATTGCACTGAAATCTAGAAATATTTTATCTGATTAATAAGATGATCTTCTTGAGATCGTTTTGGTCTGCGCGTAATCTCTTGCTCTGAAAACGGAAAAAACCGCCTTGCAGGGCGGTTTTTCGAAGGTTCTCTGAGCTACCAACTCTTTGAACCGAGGTAACTGGCTTGGAGGAGCGCAGTCACCAAAACTTGTCCTTTCAGTTTAGCCTTAACCGGCGCATGACTTCAAGACTAACTCCTCTAAATCAATTACCAGTGGCTGCTGCCAGTGGTGCTTTTGCATGTCTTTCCGGGTTGGACTCAAGACGATAGTTACCGGATAAGGCGCAGCGGTCGGACTGAACGGGGGGTTCGTGCATACAGTCCAGCTTGGAGCGAACTGCCTACCCGGAACTGAGTGTCAGGCGTGGAATGAGACAAACGCGGCCATAACAGCGGAATGACACCGGTAAACCGAAAGGCAGGAACAGGAGAGCGCACGAGGGAGCCGCCAGGGGAAACGCCTGGTATCTTTATAGTCCTGTCGGGTTTCGCCACCACTGATTTGAGCGTCAGATTTCGTGATGCTTGTCAGGGGGGCGGAGCCTATGGAAAAACGGCTTTGCCGCGGCCCTCTCACTTCCCTGTTAAGTATCTTCCTGGCATCTTCCAGGAAATCTCCGCCCCGTTCGTAAGCCATTTCCGCTCGCCGCAGTCGAACGACCGAGCGTAGCGAGTCAGTGAGCGAGGAAGCGGAATATATCCTGTATCACATATTCTGCTGACGCACCGGTGCAGCCTTTTTTCTCCTGCCACATGAAGCACTTCACTGACACCCTCATCAGTGCCAACATAGTAAGCCAGTATACACTCCGCTAGCGCTGATGTCCGGCGGTGCTTTTGCCGTTACGCACCACCCCGTCAGTAGCTGAACAGGAGGGACAGCTGATAGAAACAGAAGCCAGTTCTTTCCTGCGTTATCCCCTGATTCTGTGGATAACCGTATTACCGCCTTTGAGTGAGCTGATACCGCTCGCCGCAGCCGAACGACCGAGCGCAGCGAGTCAGTGAGCGAGGAAGCGGAAGAGCGCCTGATGCGGTATTTTCTCCTTACGCATCTGTGCGGTATTTCACACCGCATAGACCAGCCGCGTAACCTGGCAAAATCGGTTACGGTTGAGTAATAAATG-3’

---> 5V1TR: 7734bp

5’-GATGCCCTGCGTAAGCGGGTGTGGGCGGACAATAAAGTCTTAAACTGAACAAAATAGATCTAAACTATGACAATAAAGTCTTAAACTAGACAGAATAGTTGTAAACTGAAATCAGTCCAGTTATGCTGTGAAAAAGCATACTGGACTTTTGTTATGGCTAAAGCAAACTCTTCATTTTCTGAAGTGCAAATTGCCCGTCGTATTAAAGAGGGGCGTGGCCAAGGGCATGTAAAGACTATATTCGCGGCGTTGTGACAATTTACCGAACAACTCCGCGGCCGGGAAGCCGATCTCGGCTTGAACGAATTGTTAGGTGGCGGTACTTGGGTCGATATCAAAGTGCATCACTTCTTCCCGTATGCCCAACTTTGTATAGAGAGCCACTGCGGGATCGTCACCGTAATCTGCTTGCACGTAGATCACATAAGCACCAAGCGCGTTGGCCTCATGCTTGAGGAGATTGATGAGCGCGGTGGCAATGCCCTGCCTCCGGTGCTCGCCGGAGACTGCGAGATCATAGATATAGATCTCACTACGCGGCTGCTCAAACTTGGGCAGAACGTAAGCCGCGAGAGCGCCAACAACCGCTTCTTGGTCGAAGGCAGCAAGCGCGATGAATGTCTTACTACGGAGCAAGTTCCCGAGGTAATCGGAGTCCGGCTGATGTTGGGAGTAGGTGGCTACGTCTCCGAACTCACGACCGAAAAGATCAAGAGCAGCCCGCATGGATTTGACTTGGTCAGGGCCGAGCCTACATGTGCGAATGATGCCCATACTTGAGCCACCTAACTTTGTTTTAGGGCGACTGCCCTGCTGCGTAACATCGTTGCTGCTGCGTAACATCGTTGCTGCTCCATAACATCAAACATCGACCCACGGCGTAACGCGCTTGCTGCTTGGATGCCCGAGGCATAGACTGTACAAAAAAACAGTCATAACAAGCCATGAAAACCGCCACTGCGCCGTTACCACCGCTGCGTTCGGTCAAGGTTCTGGACCAGTTGCGTGAGCGCATACGCTACTTGCATTACAGTTTACGAACCGAACAGGCTTATGTCAACTGGGTTCGTGCCTTCATCCGTTTCCACGGTGTGCGTCACCCGGCAACCTTGGGCAGCAGCGAAGTCGCCATAACTTCGTATAGCATACATTATACGAAGTTATCTGTAACTATAACGGTCCTAAGGTAGCGAGTTTAAACGACGTCCTAGATTGGTTACTGGGCGATGAAGGTTTAGTCGGCAAATCGTCCAACGTTAGTGACAGCGTCAGCGGCAAGTTAATGCCTATCATTTTGTTGATAGGCGCGGTCTTGTTTTTAGGTTTAATATTTTATTTTATCTACAGATACATGATGAAAGGAGGGAAGGGAGGTGGTGTTGGCGCAGCAACGTCGCCAACTCCCATTGTTATTTCTATGCAAAACCCCACACCAACAACGGCCCCTCGATAATAAAAGACAAAAATAATATAAAATATATGTATAATTAATTAAATTCAAAATATATGTATAAGGCCGGCCTTAGTCAGTTAATTAAGTTTGTGCCCCAGTTTGCTAGGGAGGTCGCAGTATCTGGCCACAGCCACCTCGTGCTGCTCGACGTAGGTCTCTTTGTCGGCCTCCTTGATTCTTTCCAGTCTGTGGTCCACATAGTAGACGCCGGGCATCTTGAGGTTCTTAGCGGGTTTCTTGGATCTGTATGTGGTCTTGAAGTTGCAGATCAGGTGGCCCCCGCCCACGAGCTTCAGGGCCATGTCGCTTCTGCCTTCCAGGCCGCCGTCAGCGGGGTACAGCATCTCGGTGTTGGCCTCCCAGCCGAGTGTTTTCTTCTGCATCACAGGGCCGTTGGATGGGAAGTTCACCCCTCTGATCTTGACGTTGTAGATGAGGCAGCCGTCCTGGAGGCTGGTGTCCTGGGTAGCGGTCAGCACGCCCCCGTCTTCGTATGTGGTGACTCTCTCCCATGTGAAGCCCTCAGGGAAGGACTGCTTAAAGAAGTCGGGGATGCCCTGGGTGTGGTTGATGAAGGTTCTGCTGCCGTACATGAAGCTGGTAGCCAGGATGTCGAAGGCGAAGGGGAGAGGGCCGCCCTCGACCACCTTGATTCTCATGGTCTGGGTGCCCTCGTAGGGCTTGCCTTCGCCCTCGGATGTGCACTTGAAGTGGTGGTTGTTCACGGTGCCCTCCATGTACAGCTTCATGTGCATGTTCTCCTTAATCAGCTCTTCGCCCTTAGACACCATCCCGGGTGACTGGAAGTACAGGTTTTCTTGCGAGTACACCAATTCATTCATGAGTTGAGTCGCTTCCTTAACTGGCTGAAAAGGCTCTTCAGGTTTCACCATGAAAACTTTATGGCCCCCCCACAATACTGAGTCAGCATTTAATCGCCAACCACTAACCCACTGCTGCGCCTCCTGATTTGTCAACAATTCCATGAAGTTTTTCGGCACGCTTGTGAAATAATTGTTTGTGTTGGTGAAATTCGATGCTGAGTGTATACCAACAATGAACCCATCTCTAGTTGATACTAATGGACTGCCACACTGCCCATCCTTGGTTTGAATCCAATGCTTCCAGAATATGCCATCAGATGAAGGGAATGTGCAAGAAGTGTCTGACACCATGCTAGACATGCTCTTAGTTTGGAAGTTGGTTGTCACAAGACATATTCTCTCTTCCCTTTGTGGCTCTCTAAATTTCAGCTTTTGAGGAAATGGTGGGAAATCCTTAGGCATGCGAATAATTATCATGTCCCTCCCATCAATGAGGTGTTGTTGCAAAGTCGTGGTGTTCTTGACCTTGAATACACCATGTAGTGATTGGACCAACAGTGTTCCATTATTTCTTCTAAACAAGTGCTTGTTTGTAATGATGAAGGGACCAAATCCAATACCATACAACGATGTTGTGTGCCCATCAGATTCATTCGTCAAATGACAAATGGTGCTCGATATAGGGTTGTAATCACGCGGCCCCTTAAACAAGCTCTCTCCGGCGTAGTCAGGCACGTCGTAAGGATAAGCCATATTTAAATATATGCTTGCTTGTGTGTTCCTTATTGAAGCCTTGGTGTGACTGATTTACTAGTAGCGTTGAGGCGTCTTATATACCCGACCGTTATCTGGCCTACGTGACACAAGGCACGTTGTTAGATTAATAATCTTATCTTTTTATCTTAATTGATAAGATTATTTTTATCTGGCTGTTATAAAAACGGGATCATGAACACGGACGCTCAGTCGACAGATCTGTCGACGGTTTAAACACTAGTTCGCGACCTACTCCGGAATATTAATAGGTTGCTGATATCGGGAGTTCAGTCGTCGAATGCAAAGCGTAAAAAATATTAATAAGGTAAAAATTACAGCTACATAAATTACACAATTTAAACGGATCGATGAGCTCCATATGGCCTGGAGCCATCCGCAATTTGAAAAAGGTGGCGGGTCCGGCGGAGGTAGCGGCGGAGGTTCTTGGTCTCACCCTCAGTTCGAGAAGGATGACGATGATAAAACCATGGGATCCCTAGGTACCGCGGCCGCGCGCGTTGGCCGATTCATTAATGCAGCTGGCACGACAGGTTTCCCGACTGGAAAGCGGGCAGTGAGCGCAACGCAATTAATGTGAGTTAGCTCACTCATTAGGCACCCCAGGCTTTACACTTTATGCTTCCGGCTCGTATGTTGTGTGGAATTGTGAGCGGATAACAATTTCACACAGGAAACAGCTATGACCATGATTACGCCAAGCTATTTAGGTGACGCGTTAGAATACTCAAGCTATGCATCATCTTTGGTTCCGTCATCGGACCCATTAGTAACGGCCGCCAGTGTGCTGGAGTTTTGTAGATACCCATCACACTGGCGTCCACTGGAACATGCAAGTAGAGGGCCCAATTCGCCCTATAGTGAGTCGTATTACAATTCACTGGCCGTCGTTTTACAACGTCGTGACTGGGAAAACCCTGGCGTTACCCAACTTAATCGCCTTGCAGCACATCCCCCTTTCGCCAGCTGGCGTAATAGCGAAGAGGCCCGCACCGATCGCCCTTCCCAACAGTTGCGCAGCCTATACGTACGGTAACTGACTAAGAATTCCTTAAGCGGAGGCCTGCAGGGCTCGAGTTAACTGACTAAAAGCTTCGAAAGGAAGCTGAGTTGGCTGCTGCCACCGCTGAGCAATAACTAGCATAACCCCTTGGGGCCTCTAAACGGGTCTTGAGGGGTTTTTTGCTGAAAGGAGGAACTATCCTCAGGGTCGAGAAGTACTAGAGGATCATAATCAGCCATACCACATTTGTAGAGGTTTTACTTGCTTTAAAAAACCTCCCACACCTCCCCCTGAACCTGAAACATAAAATGAATGCAATTGTTGTTGTTAACTTGTTTATTGCAGCTTATAATGGTTACAAATAAAGCAATAGCATCACAAATTTCACAAATAAAGCATTTTTTTCACTGCATTCTAGTTGTGGTTTGTCCAAACTCATCAATGTATCTTATCATGTCTGGATCTGATCACTGCTTGAGCCTAGGAGATCCGAACCAGATAAGTGAAATCTAGTTCCAAACTATTTTGTCATTTTTAATTTTCGTATTAGCTTACGACGCTACACCCAGTTCCCATCTATTTTGTCACTCTTCCCTAAATAATCCTTAAAAACTCCATTTCCACCCCTCCCAGTTCCCAACTATTTTGTCCGCCCACAGCGGGGCATTTTTCTTCCTGTTATGTTTTTAATCAAACATCCTGCCAACTCCATGTGACAAACCGTCATCTTCGGCTACTTTTTCTCTGTCACAGAATGAAAATTTTTCTGTCATCTCTTCGTTATTAATGTTTGTAATTGACTGAATATCAACGCTTATTTGCAGCCTGAATGGCGAATGGGACGCGCCCTGTAGCGGCGCATTAAGCGCGGCGGGTGTGGTGGTTACGCGCAGCGTGACCGCTACACTTGCCAGCGCCCTAGCGCCCGCTCCTTTCGCTTTCTTCCCTTCCTTTCTCGCCACGTTCGCCGGCTTTCCCCGTCAAGCTCTAAATCGGGGGCTCCCTTTAGGGTTCCGATTTAGTGCTTTACGGCACCTCGACCCCAAAAAACTTGATTAGGGTGATGGTTCACGTAGTGGGCCATCGCCCTGATAGACGGTTTTTCGCCCTTTGACGTTGGAGTCCACGTTCTTTAATAGTGGACTCTTGTTCCAAACTGGAACAACACTCAACCCTATCTCGGTCTATTCTTTTGATTTATAAGGGATTTTGCCGATTTCGGCCTATTGGTTAAAAAATGAGCTGATTTAACAAAAATTTAACGCGAATTTTAACAAAATATTAACGTTTACAATTTCAGGTGGCACTTTTCGGGGAAATGTGCGCGGAACCCCTATTTGTTTATTTTTCTAAATACATTCAAATATGTATCCGCTCATGAGACAATAACCCTGATAAATGCTTCAATAATATTGAAAAAGGAAGAGTATGAGTATTCAACATTTCCGTGTCGCCCTTATTCCCTTTTTTGCGGCATTTTGCCTTCCTGTTTTTGCTCACCCAGAAACGCTGGTGAAAGTAAAAGATGCTGAAGATCAGTTGGGTGCACGAGTGGGTTACATCGAACTGGATCTCAACAGCGGTAAGATCCTTGAGAGTTTTCGCCCCGAAGAACGTTTTCCAATGATGAGCACTTTTAAAGTTCTGCTATGTGGCGCGGTATTATCCCGTATTGACGCCGGGCAAGAGCAACTCGGTCGCCGCATACACTATTCTCAGAATGACTTGGTTGAGTACTCACCAGTCACAGAAAAGCATCTTACGGATGGCATGACAGTAAGAGAATTATGCAGTGCTGCCATAACCATGAGTGATAACACTGCGGCCAACTTACTTCTGACAACGATCGGAGGACCGAAGGAGCTAACCGCTTTTTTGCACAACATGGGGGATCATGTAACTCGCCTTGATCGTTGGGAACCGGAGCTGAATGAAGCCATACCAAACGACGAGCGTGACACCACGATGCCTGTAGCAATGGCAACAACGTTGCGCAAACTATTAACTGGCGAACTACTTACTCTAGCTTCCCGGCAACAATTAATAGACTGGATGGAGGCGGATAAAGTTGCAGGACCACTTCTGCGCTCGGCCCTTCCGGCTGGCTGGTTTATTGCTGATAAATCTGGAGCCGGTGAGCGTGGGTCTCGCGGTATCATTGCAGCACTGGGGCCAGATGGTAAGCCCTCCCGTATCGTAGTTATCTACACGACGGGGAGTCAGGCAACTATGGATGAACGAAATAGACAGATCGCTGAGATAGGTGCCTCACTGATTAAGCATTGGTAACTGTCAGACCAAGTTTACTCATATATACTTTAGATTGATTTAAAACTTCATTTTTAATTTAAAAGGATCTAGGTGAAGATCCTTTTTGATAATCTCATGACCAAAATCCCTTAACGTGAGTTTTCGTTCCACTGAGCGTCAGACCGCGGGGCATGACTAACATGAGAATTACAACTTATATCGTATGGGGCTGACTTCAGGTGCTACATTTGAAGAGATAAATTGCACTGAAATCTAGAAATATTTTATCTGATTAATAAGATGATCTTCTTGAGATCGTTTTGGTCTGCGCGTAATCTCTTGCTCTGAAAACGGAAAAAACCGCCTTGCAGGGCGGTTTTTCGAAGGTTCTCTGAGCTACCAACTCTTTGAACCGAGGTAACTGGCTTGGAGGAGCGCAGTCACCAAAACTTGTCCTTTCAGTTTAGCCTTAACCGGCGCATGACTTCAAGACTAACTCCTCTAAATCAATTACCAGTGGCTGCTGCCAGTGGTGCTTTTGCATGTCTTTCCGGGTTGGACTCAAGACGATAGTTACCGGATAAGGCGCAGCGGTCGGACTGAACGGGGGGTTCGTGCATACAGTCCAGCTTGGAGCGAACTGCCTACCCGGAACTGAGTGTCAGGCGTGGAATGAGACAAACGCGGCCATAACAGCGGAATGACACCGGTAAACCGAAAGGCAGGAACAGGAGAGCGCACGAGGGAGCCGCCAGGGGAAACGCCTGGTATCTTTATAGTCCTGTCGGGTTTCGCCACCACTGATTTGAGCGTCAGATTTCGTGATGCTTGTCAGGGGGGCGGAGCCTATGGAAAAACGGCTTTGCCGCGGCCCTCTCACTTCCCTGTTAAGTATCTTCCTGGCATCTTCCAGGAAATCTCCGCCCCGTTCGTAAGCCATTTCCGCTCGCCGCAGTCGAACGACCGAGCGTAGCGAGTCAGTGAGCGAGGAAGCGGAATATATCCTGTATCACATATTCTGCTGACGCACCGGTGCAGCCTTTTTTCTCCTGCCACATGAAGCACTTCACTGACACCCTCATCAGTGCCAACATAGTAAGCCAGTATACACTCCGCTAGCGCTGATGTCCGGCGGTGCTTTTGCCGTTACGCACCACCCCGTCAGTAGCTGAACAGGAGGGACAGCTGATAGAAACAGAAGCCAGTTCTTTCCTGCGTTATCCCCTGATTCTGTGGATAACCGTATTACCGCCTTTGAGTGAGCTGATACCGCTCGCCGCAGCCGAACGACCGAGCGCAGCGAGTCAGTGAGCGAGGAAGCGGAAGAGCGCCTGATGCGGTATTTTCTCCTTACGCATCTGTGCGGTATTTCACACCGCATAGACCAGCCGCGTAACCTGGCAAAATCGGTTACGGTTGAGTAATAAATG-3’

---> 4V2G: 4915bp

5’-CGCGCCGGTATGTACAGGAAGAGGTTTATACTAAACTGTTACATTGCAAACGTGGTTTCGTGTGCCAAGTGTGAAAACCGATGTTTAATCAAGGCTCTGACGCATTTCTACAACCACGACTCCAAGTGTGTGGGTGAAGTCAGATGTTTAAACCCATGTGCCTGGCAGATAACTTCGTATAATGTATGCTATACGAAGTTATGGTACGCGGCCGCGTAGAGGATCTGTTGATCAGCAGTTCAACCTGTTGATAATACGGACCTTTAATTCAACCCAACACAATATATTATAGTTAAATAAGAATTATTATCAAATCATTTGTATATTAATTAAAATACTATACTGTAAATTACATTTTATTTACAATCACTCGACACCGGTGATATCCATATGGCACATCACCACCATCATCACCATCACCACCACGGAGGCAGCGATGACGATGATAAAACCATGGGATCCGCTAGCTTAAGCTGTCAGACCAAGTTTACTCATATATACTTTAGATTGATTTAAAACTTCATTTTTAATTTAAAAGGATCTAGGTGAAGATCCTTTTTGATAATCTCATGACCAAAATCCCTTAACGTGAGTTTTCGTTCCACTGAGCGTCAGACCCCGTAGAAAAGATCAAAGGATCTTCTTGAGATCCTTTTTTTCTGCGCGTAATCTGCTGCTTGCAAACAAAAAAACCACCGCTACCAGCGGTGGTTTGTTTGCCGGATCAAGAGCTACCAACTCTTTTTCCGAAGGTAACTGGCTTCAGCAGAGCGCAGATACCAAATACTGTTCTTCTAGTGTAGCCGTAGTTAGGCCACCACTTCAAGAACTCTGTAGCACCGCCTACATACCTCGCTCTGCTAATCCTGTTACCAGTGGCTGCTGCCAGTGGCGATAAGTCGTGTCTTACCGGGTTGGACTCAAGACGATAGTTACCGGATAAGGCGCAGCGGTCGGGCTGAACGGGGGGTTCGTGCACACAGCCCAGCTTGGAGCGAACGACCTACACCGAACTGAGATACCTACAGCGTGAGCTATGAGAAAGCGCCACGCTTCCCGAAGGGAGAAAGGCGGACAGGTATCCGGTAAGCGGCAGGGTCGGAACAGGAGAGCGCACGAGGGAGCTTCCAGGGGGAAACGCCTGGTATCTTTATAGTCCTGTCGGGTTTCGCCACCTCTGACTTGAGCGTCGATTTTTGTGATGCTCGTCAGGGGGGCGGAGCCTATGGAAAAACGCCAGCAACGCGGCCTTTTTACGGTTCCTGGCCTTTTGCTGGCCTTTTGCTCACATGTTCTTTCCTGCGTTATCCCCTGATTCTGTGGATAACCGTATTACCGCCTTTGAGTGAGCTGATACCGCTCGCCGCAGCCGAACGACCGAGCGCAGCGAGTCAGTGAGCGAGGAAGCGGAAGAGCGCCCAATACGCAAACCGCCTCTCCCCGCGCGTTGGCCGATTCATTAATGCAGCTGGCACGACAGGTTTCCCGACTGGAAAGCGGGCAGTGAGCGCAACGCAATTAATGTGAGTTAGCTCACTCATTAGGCACCCCAGGCTTTACACTTTATGCTTCCGGCTCGTATGTTGTGTGGAATTGTGAGCGGATAACAATTTCACACAGGAAACAGCTATGACCATGATTACGCCAAGCTATTTAGGTGACGCGTTAGAATACTCAAGCTATGCATCATCTTTGGTTCCGTCATCGGACCCATTAGTAACGGCCGCCAGTGTGCTGGAGTTTTGTAGATACCCATCACACTGGCGTCCACTGGAACATGCAAGTAGAGGGCCCAATTCGCCCTATAGTGAGTCGTATTACAATTCACTGGCCGTCGTTTTACAACGTCGTGACTGGGAAAACCCTGGCGTTACCCAACTTAATCGCCTTGCAGCACATCCCCCTTTCGCCAGCTGGCGTAATAGCGAAGAGGCCCGCACCGATCGCCCTTCCCAACAGTTGCGCAGCCTATACGTACGGTAACTGACTAAGAATTCCGATTACAAAGACGATGACGACAAGGGCTCGAGTGAAAATTTGTATTTTCAAAGCGGGAGCTCTGTGAGCAAGGGCGAGGAGCTGTTCACCGGGGTGGTGCCCATCCTGGTCGAGCTGGACGGCGACGTAAACGGCCACAAGTTCAGCGTGTCCGGCGAGGGCGAGGGCGATGCCACCTACGGCAAGCTGACCCTGAAGTTCATCTGCACCACCGGCAAGCTGCCCGTGCCCTGGCCCACCCTCGTGACCACCCTGACCTACGGCGTGCAGTGCTTCAGCCGCTACCCCGACCACATGAAGCAGCACGACTTCTTCAAGTCCGCCATGCCCGAAGGCTACGTCCAGGAGCGCACCATCTTCTTCAAGGACGACGGCAACTACAAGACCCGCGCCGAGGTGAAGTTCGAGGGCGACACCCTGGTGAACCGCATCGAGCTGAAGGGCATCGACTTCAAGGAGGACGGCAACATCCTGGGGCACAAGCTGGAGTACAACTACAACAGCCACAACGTCTATATCATGGCCGACAAGCAGAAGAACGGCATCAAGGTGAACTTCAAGATCCGCCACAACATCGAGGACGGCAGCGTGCAGCTCGCCGACCACTACCAGCAGAACACCCCCATCGGCGACGGCCCCGTGCTGCTGCCCGACAACCACTACCTGAGCACCCAGTCCGCCCTGAGCAAAGACCCCAACGAGAAGCGCGATCACATGGTCCTGCTGGAGTTCGTGACCGCCGCCGGGATCACTCTCGGCATGGACGAGCTGTACAAGTAACTGACTAAGGTACCCGAAAGGAAGCTGAGTTGGCTGCTGCCACCGCTGAGCAATAACTAGCATAACCCCTTGGGGCCTCTAAACGGGTCTTGAGGGGTTTTTTGCTGAAAGGAGGAACTATCCTCAGGGGGAGATGGGGGAGGCTAACTGAAACACGGAAGGAGACAATACCGGAAGGAACCCGCGCTATGACGGCAATAAAAAGACAGAATAAAACGCACGGGTGTTGGGTCGTTTGTTCATAAACGCGGGGTTCGGTCCCAGGGCTGGCACTCTGTCGATACCCCACCGAGACCCCATTGGGACCAATACGCCCGCGTTTCTTCCTTTTCCCCACCCCAACCCCCAAGTTCGGGTGAAGGCCCAGGGCTCGCAGCCAACGTCGGGGCGGCAAGCCCTGCCATAGCCACTACGGGTACGTCTGAAAGCATGCCTTTTTGGAATTTACGTACTAAGCTCTCATGTTTCACGTACTAAGCTCTCATGTTTAACGTACTAAGCTCTCATGTTTAACGAACTAAACCCTCATGGCTAACGTACTAAGCTCTCATGGCTAACGTACTAAGCTCTCATGTTTCACGTACTAAGCTCTCATGTTTGAACAATAAAATTAATATAAATCAGCAACTTAAATAGCCTCTAAGGTTTTAAGTTTTATAAGAAAAAAAAGAATATATAAGGCTTTTAAAGCTTTTAAGGTTTAACGGTTGTGGACAACAAGCCAGGGATGTAACGCACTGAGAAGCCCTTAGAGCCTCTCAAAGCAATTTTCAGTGACACAGGAACACTTAACGGCTGACAGAATTAGCTTCACGCTGCCGCAAGCACTCAGGGCGCAAGGGCTGCTAAAGGAAGCGGAACACGTAGAAAGCCAGTCCGCAGAAACGGTGCTGACCCCGGATGAATGTCAGCTACTGGGCTATCTGGACAAGGGAAAACGCAAGCGCAAAGAGAAAGCAGGTAGCTTGCAGTGGGCTTACATGGCGATAGCTAGACTGGGCGGTTTTATGGACAGCAAGCGAACCGGAATTGCCAGCTGGGGCGCCCTCTGGTAAGGTTGGGAAGCCCTGCAAAGTAAACTGGATGGCTTTCTTGCCGCCAAGGATCTGATGGCGCAGGGGATCAAGATCTGATCAAGAGACAGGATGAGGATCGTTTCGCATGATTGAACAAGATGGATTGCACGCAGGTTCTCCGGCCGCTTGGGTGGAGAGGCTATTCGGCTATGACTGGGCACAACAGACAATCGGCTGCTCTGATGCCGCCGTGTTCCGGCTGTCAGCGCAGGGGCGCCCGGTTCTTTTTGTCAAGACCGACCTGTCCGGTGCCCTGAATGAACTGCAGGACGAGGCAGCGCGGCTATCGTGGCTGGCCACGACGGGCGTTCCTTGCGCAGCTGTGCTCGACGTTGTCACTGAAGCGGGAAGGGACTGGCTGCTATTGGGCGAAGTGCCGGGGCAGGATCTCCTGTCATCTCACCTTGCTCCTGCCGAGAAAGTATCCATCATGGCTGATGCAATGCGGCGGCTGCATACGCTTGATCCGGCTACCTGCCCATTCGACCACCAAGCGAAACATCGCATCGAGCGAGCACGTACTCGGATGGAAGCCGGTCTTGTCGATCAGGATGATCTGGACGAAGAGCATCAGGGGCTCGCGCCAGCCGAACTGTTCGCCAGGCTCAAGGCGCGCATGCCCGACGGCGAGGATCTCGTCGTGACACATGGCGATGCCTGCTTGCCGAATATCATGGTGGAAAATGGCCGCTTTTCTGGATTCATCGACTGTGGCCGGCTGGGTGTGGCGGACCGCTATCAGGACATAGCGTTGGCTACCCGTGATATTGCTGAAGAGCTTGGCGGCGAATGGGCTGACCGCTTCCTCGTGCTTTACGGTATCGCCGCTCCCGATTCGCAGCGCATCGCCTTCTATCGCCTTCTTGACGAGTTCTTCTGAGCGGGACTCTGGGGTTCGAAATGACCGACCAAGCGACGCCCAACCTGCCATCACGAGATTTCGATTCCACCGCCGCCTTCTATGAAAGGTTGGGCTTCGGAATCGTTTTCCGGGACGCCGGCTGGATGATCCTCCAGCGCGGGGATCTCATGCTGGAGTTCTTCGCCCACATCATATCGAT-3’

---> 4V2R: 4915bp

5’-CGCGCCGGTATGTACAGGAAGAGGTTTATACTAAACTGTTACATTGCAAACGTGGTTTCGTGTGCCAAGTGTGAAAACCGATGTTTAATCAAGGCTCTGACGCATTTCTACAACCACGACTCCAAGTGTGTGGGTGAAGTCAGATGTTTAAACCCATGTGCCTGGCAGATAACTTCGTATAATGTATGCTATACGAAGTTATGGTACGCGGCCGCGTAGAGGATCTGTTGATCAGCAGTTCAACCTGTTGATAATACGGACCTTTAATTCAACCCAACACAATATATTATAGTTAAATAAGAATTATTATCAAATCATTTGTATATTAATTAAAATACTATACTGTAAATTACATTTTATTTACAATCACTCGACACCGGTGATATCCATATGGCACATCACCACCATCATCACCATCACCACCACGGAGGCAGCGATGACGATGATAAAACCATGGGATCCGCTAGCTTAAGCTGTCAGACCAAGTTTACTCATATATACTTTAGATTGATTTAAAACTTCATTTTTAATTTAAAAGGATCTAGGTGAAGATCCTTTTTGATAATCTCATGACCAAAATCCCTTAACGTGAGTTTTCGTTCCACTGAGCGTCAGACCCCGTAGAAAAGATCAAAGGATCTTCTTGAGATCCTTTTTTTCTGCGCGTAATCTGCTGCTTGCAAACAAAAAAACCACCGCTACCAGCGGTGGTTTGTTTGCCGGATCAAGAGCTACCAACTCTTTTTCCGAAGGTAACTGGCTTCAGCAGAGCGCAGATACCAAATACTGTTCTTCTAGTGTAGCCGTAGTTAGGCCACCACTTCAAGAACTCTGTAGCACCGCCTACATACCTCGCTCTGCTAATCCTGTTACCAGTGGCTGCTGCCAGTGGCGATAAGTCGTGTCTTACCGGGTTGGACTCAAGACGATAGTTACCGGATAAGGCGCAGCGGTCGGGCTGAACGGGGGGTTCGTGCACACAGCCCAGCTTGGAGCGAACGACCTACACCGAACTGAGATACCTACAGCGTGAGCTATGAGAAAGCGCCACGCTTCCCGAAGGGAGAAAGGCGGACAGGTATCCGGTAAGCGGCAGGGTCGGAACAGGAGAGCGCACGAGGGAGCTTCCAGGGGGAAACGCCTGGTATCTTTATAGTCCTGTCGGGTTTCGCCACCTCTGACTTGAGCGTCGATTTTTGTGATGCTCGTCAGGGGGGCGGAGCCTATGGAAAAACGCCAGCAACGCGGCCTTTTTACGGTTCCTGGCCTTTTGCTGGCCTTTTGCTCACATGTTCTTTCCTGCGTTATCCCCTGATTCTGTGGATAACCGTATTACCGCCTTTGAGTGAGCTGATACCGCTCGCCGCAGCCGAACGACCGAGCGCAGCGAGTCAGTGAGCGAGGAAGCGGAAGAGCGCCCAATACGCAAACCGCCTCTCCCCGCGCGTTGGCCGATTCATTAATGCAGCTGGCACGACAGGTTTCCCGACTGGAAAGCGGGCAGTGAGCGCAACGCAATTAATGTGAGTTAGCTCACTCATTAGGCACCCCAGGCTTTACACTTTATGCTTCCGGCTCGTATGTTGTGTGGAATTGTGAGCGGATAACAATTTCACACAGGAAACAGCTATGACCATGATTACGCCAAGCTATTTAGGTGACGCGTTAGAATACTCAAGCTATGCATCATCTTTGGTTCCGTCATCGGACCCATTAGTAACGGCCGCCAGTGTGCTGGAGTTTTGTAGATACCCATCACACTGGCGTCCACTGGAACATGCAAGTAGAGGGCCCAATTCGCCCTATAGTGAGTCGTATTACAATTCACTGGCCGTCGTTTTACAACGTCGTGACTGGGAAAACCCTGGCGTTACCCAACTTAATCGCCTTGCAGCACATCCCCCTTTCGCCAGCTGGCGTAATAGCGAAGAGGCCCGCACCGATCGCCCTTCCCAACAGTTGCGCAGCCTATACGTACGGTAACTGACTAAGAATTCCGAGCAAAAGTTGATTAGCGAAGAAGACTTAGGCTCGAGTGAAAATTTGTATTTTCAAAGCGGGAGCTCTGTGTCTAAGGGCGAAGAGCTGATTAAGGAGAACATGCACATGAAGCTGTACATGGAGGGCACCGTGAACAACCACCACTTCAAGTGCACATCCGAGGGCGAAGGCAAGCCCTACGAGGGCACCCAGACCATGAGAATCAAGGTGGTCGAGGGCGGCCCTCTCCCCTTCGCCTTCGACATCCTGGCTACCAGCTTCATGTACGGCAGCAGAACCTTCATCAACCACACCCAGGGCATCCCCGACTTCTTTAAGCAGTCCTTCCCTGAGGGCTTCACATGGGAGAGAGTCACCACATACGAAGACGGGGGCGTGCTGACCGCTACCCAGGACACCAGCCTCCAGGACGGCTGCCTCATCTACAACGTCAAGATCAGAGGGGTGAACTTCCCATCCAACGGCCCTGTGATGCAGAAGAAAACACTCGGCTGGGAGGCCAACACCGAGATGCTGTACCCCGCTGACGGCGGCCTGGAAGGCAGAAGCGACATGGCCCTGAAGCTCGTGGGCGGGGGCCACCTGATCTGCAACTTCAAGACCACATACAGATCCAAGAAACCCGCTAAGAACCTCAAGATGCCCGGCGTCTACTATGTGGACCACAGACTGGAAAGAATCAAGGAGGCCGACAAAGAGACCTACGTCGAGCAGCACGAGGTGGCTGTGGCCAGATACTGCGACCTCCCTAGCAAACTGGGGCACAAACTTAATTAACTGACTAAGGTACCCGAAAGGAAGCTGAGTTGGCTGCTGCCACCGCTGAGCAATAACTAGCATAACCCCTTGGGGCCTCTAAACGGGTCTTGAGGGGTTTTTTGCTGAAAGGAGGAACTATCCTCAGGGGGAGATGGGGGAGGCTAACTGAAACACGGAAGGAGACAATACCGGAAGGAACCCGCGCTATGACGGCAATAAAAAGACAGAATAAAACGCACGGGTGTTGGGTCGTTTGTTCATAAACGCGGGGTTCGGTCCCAGGGCTGGCACTCTGTCGATACCCCACCGAGACCCCATTGGGACCAATACGCCCGCGTTTCTTCCTTTTCCCCACCCCAACCCCCAAGTTCGGGTGAAGGCCCAGGGCTCGCAGCCAACGTCGGGGCGGCAAGCCCTGCCATAGCCACTACGGGTACGTCTGAAAGCATGCCTTTTTGGAATTTACGTACTAAGCTCTCATGTTTCACGTACTAAGCTCTCATGTTTAACGTACTAAGCTCTCATGTTTAACGAACTAAACCCTCATGGCTAACGTACTAAGCTCTCATGGCTAACGTACTAAGCTCTCATGTTTCACGTACTAAGCTCTCATGTTTGAACAATAAAATTAATATAAATCAGCAACTTAAATAGCCTCTAAGGTTTTAAGTTTTATAAGAAAAAAAAGAATATATAAGGCTTTTAAAGCTTTTAAGGTTTAACGGTTGTGGACAACAAGCCAGGGATGTAACGCACTGAGAAGCCCTTAGAGCCTCTCAAAGCAATTTTCAGTGACACAGGAACACTTAACGGCTGACAGAATTAGCTTCACGCTGCCGCAAGCACTCAGGGCGCAAGGGCTGCTAAAGGAAGCGGAACACGTAGAAAGCCAGTCCGCAGAAACGGTGCTGACCCCGGATGAATGTCAGCTACTGGGCTATCTGGACAAGGGAAAACGCAAGCGCAAAGAGAAAGCAGGTAGCTTGCAGTGGGCTTACATGGCGATAGCTAGACTGGGCGGTTTTATGGACAGCAAGCGAACCGGAATTGCCAGCTGGGGCGCCCTCTGGTAAGGTTGGGAAGCCCTGCAAAGTAAACTGGATGGCTTTCTTGCCGCCAAGGATCTGATGGCGCAGGGGATCAAGATCTGATCAAGAGACAGGATGAGGATCGTTTCGCATGATTGAACAAGATGGATTGCACGCAGGTTCTCCGGCCGCTTGGGTGGAGAGGCTATTCGGCTATGACTGGGCACAACAGACAATCGGCTGCTCTGATGCCGCCGTGTTCCGGCTGTCAGCGCAGGGGCGCCCGGTTCTTTTTGTCAAGACCGACCTGTCCGGTGCCCTGAATGAACTGCAGGACGAGGCAGCGCGGCTATCGTGGCTGGCCACGACGGGCGTTCCTTGCGCAGCTGTGCTCGACGTTGTCACTGAAGCGGGAAGGGACTGGCTGCTATTGGGCGAAGTGCCGGGGCAGGATCTCCTGTCATCTCACCTTGCTCCTGCCGAGAAAGTATCCATCATGGCTGATGCAATGCGGCGGCTGCATACGCTTGATCCGGCTACCTGCCCATTCGACCACCAAGCGAAACATCGCATCGAGCGAGCACGTACTCGGATGGAAGCCGGTCTTGTCGATCAGGATGATCTGGACGAAGAGCATCAGGGGCTCGCGCCAGCCGAACTGTTCGCCAGGCTCAAGGCGCGCATGCCCGACGGCGAGGATCTCGTCGTGACACATGGCGATGCCTGCTTGCCGAATATCATGGTGGAAAATGGCCGCTTTTCTGGATTCATCGACTGTGGCCGGCTGGGTGTGGCGGACCGCTATCAGGACATAGCGTTGGCTACCCGTGATATTGCTGAAGAGCTTGGCGGCGAATGGGCTGACCGCTTCCTCGTGCTTTACGGTATCGCCGCTCCCGATTCGCAGCGCATCGCCTTCTATCGCCTTCTTGACGAGTTCTTCTGAGCGGGACTCTGGGGTTCGAAATGACCGACCAAGCGACGCCCAACCTGCCATCACGAGATTTCGATTCCACCGCCGCCTTCTATGAAAGGTTGGGCTTCGGAATCGTTTTCCGGGACGCCGGCTGGATGATCCTCCAGCGCGGGGATCTCATGCTGGAGTTCTTCGCCCACATCATATCGAT-3’

**S2. Degenerate nucleotide sequences of the TCS cleavage sites used in the Smartbac system**

Sequence 1: 5’-GAAAACCTGTACTTCCAGTCA-3’

Sequence 2: 5’-GAAAATTTGTATTTTCAAAGC-3’

Sequence 3: 5’-GAGAACTTGTACTTTCAGAGT-3’

Sequence 4: 5’-GAGAATCTGTATTTCCAGAGC-3’

Sequence 5: 5’-GAAAACTTGTATTTCCAATCT-3’

Sequence 6: 5’-GAGAATTTATACTTTCAATCC-3’

Sequence 7: 5’-GAGAACTTATATTTCCAGTCA-3’

Sequence 8: 5’-GAGAACCTCTACTTCCAATCC-3’

Sequence 9: 5’-GAGAACCTGTATTTTCAGAGC-3’

Sequence 10: 5’-GAGAACCTGTACTTCCAGTCA-3’

**S3. Primer sequences used to construct the clone of the human exocyst complex**

E1F1：5’- TATCCATATGGGATCCACAGCAATCAAGCATGCATTACAAAG-3’

E1F2: 5’- ATCACTCGACACCGGTGATATCCATATGGGATCCACAGCAATCAAG-3’

E1R1: 5’- CAGGTTTTCACTCGAGCCGTGGGACTGTGCAATGCTGGAACAATAATC-3’

E1R2: 5’- TAAGCTAGAGCTCTGGAAGTACAGGTTTTCACTCGAGCCGTGGGACTG-3’

E5F1: 5’-GCTTAAGCGCGGCCGCGACCACGGCCGAGTTGTTCGAGGAGCCTTTTG-3’

E5F2: 5’-ACCTGTACTTCCAGAGCTCTAGCTTAAGCGCGGCCGCGACCACGGCCG-3’

E5R1: 5’-CCTTTCGGGTACCCTCGAGTTAGCTGAAGTGTCGAGCAAGGCGGGCAG-3’

E5R2: 5’-CAGCAGCCAACTCAGCTTCCTTTCGGGTACCCTCGAGTTAGCTG-3’

E2F1: 5’-TATCCATATGGCGGCCGCTAGCCGATCACGACAACCCCCCCTTG-3’

E2F2: 5’- ATCACTCGACACCGGTGATATCCATATGGCGGCCGCTAGCCGATC-3’

E2R1: 5’-ATACAAATTTTCACTCGAGCCTGTTTTCATCATGGTTGAAGAAGCTGC-3’

E2R2: 5’- GTACCACTAGTGCTTTGAAAATACAAATTTTCACTCGAGCCTGTTTTC-3’

E8F1: 5’-AGCACTAGTGGTACCCTTAAGATGGCGATGGCGATGTCGGACAGTGGG-3’

E8F2: 5’-AGTGAAAATTTGTATTTTCAAAGCACTAGTGGTACCCTTAAGATGGCG-3’

E3F1: 5’-AGAGCGGTACCGCGGCCGCGATGAAGGAGACAGACCGGGAGGCCGTTG-3’

E3F2: 5’-CGAGTGAGAATCTGTATTTCCAGAGCGGTACCGCGGCCGCGATGAAGG-3’

E6F1: 5’-AGCTCCATATGGGATCCGCGGAGAACAGCGAGAGTCTGGGCAC-3’

E6F2: 5’-ATTTAAACGGATCGATGAGCTCCATATGGGATCCGCGGAGAAC-3’

E6R1: 5’-ACAGATTCTCACTCGAGCCCATGTGCTGGGACATACCATTCACCAAAC-3’

E6R2: 5’-CGCGGTACCGCTCTGGAAATACAGATTCTCACTCGAGCCCATGTGCTG-3’

E4F1: 5’-GCTCCATATGGCGGCCGCGGAAGCAGCTGGTGGGAAATACAGAAG-3’

E4F2: 5’-ATTTAAACGGATCGATGAGCTCCATATGGCGGCCGCGGAAGCAGC-3’

E4R1: 5’-AGTACAAGTTCTCACTCGAGCCAACGGTAGTTATCTTCTTGTCCTTGG-3’

E4R2: 5’-GTACCGCTTAAGGACTGAAAGTACAAGTTCTCACTCGAGCCAACGG-3’

E7F1: 5’-TCAGTCCTTAAGCGGTACCATGATTCCCCCACAGGAGGCATCCGCTC-3’

E7F2: 5’-AGTGAGAACTTGTACTTTCAGTCCTTAAGCGGTACCATGATTCC-3’

E7R1: 5’-TCAGTTAACTCGAGTTAGGCAGAGGTGTCAAAAAGGCGATCG-3’

E7R2: 5’-CCTTTCGAAGCTTTTAGTCAGTTAACTCGAGTTAGGCAGAGGTG-3’

S5-F: 5’-TACTTCCAGAGCTCTAGCTTAAGCATGGCCTGGAGCCATCCGCAATTTG-3’

S5-R: 5’-CAACTCAGCTTCCTTTCGGGTACCTTAGCTGAAGTGTCGAGCAAGGCGG-3’
